# A review on COVID-19 transmission, epidemiological features, prevention and vaccination

**DOI:** 10.1515/mr-2021-0023

**Published:** 2022-03-02

**Authors:** Yuqin Zhang, Gonghua Wu, Shirui Chen, Xu Ju, Wumitijiang Yimaer, Wangjian Zhang, Shao Lin, Yuantao Hao, Jing Gu, Jinghua Li

**Affiliations:** School of Public Health, Sun Yat-Sen University, Guangzhou, China; Department of Environmental Health Sciences, School of Public Health, University at Albany, State University of New York, Rensselaer, NY, USA; Sun Yat-Sen University Global Health Institute, School of Public Health and Institute of State Governance, Sun Yat-Sen University, Guangzhou, China

**Keywords:** coronavirus, COVID-19, pandemic, review, SARS-CoV-2

## Abstract

Severe acute respiratory syndrome coronavirus 2 (SARS-CoV-2) has caused hundreds of millions of infections and millions of deaths over past two years. Currently, many countries have still not been able to take the pandemic under control. In this review, we systematically summarized what we have done to mitigate the COVID-19 pandemic, from the perspectives of virus transmission, public health control measures, to the development and vaccination of COVID-19 vaccines. As a virus most likely coming from bats, the SARS-CoV-2 may transmit among people via airborne, faecal-oral, vertical or foodborne routes. Our meta-analysis suggested that the R_0_ of COVID-19 was 2.9 (95% CI: 2.7–3.1), and the estimates in Africa and Europe could be higher. The median R_t_ could decrease by 23–96% following the nonpharmacological interventions, including lockdown, isolation, social distance, and face mask, etc. Comprehensive intervention and lockdown were the most effective measures to control the pandemic. According to the pooled R_0_ in our meta-analysis, there should be at least 93.3% (95% CI: 89.9–96.2%) people being vaccinated around the world. Limited amount of vaccines and the inequity issues in vaccine allocation call for more international cooperation to achieve the anti-epidemic goals and vaccination fairness.

## Introduction

Three major severe epidemics have been reported over the past two decades, including Severe Acute Respiratory Syndrome (SARS), Middle East Respiratory Syndrome (MERS), and the latest severe acute respiratory coronavirus syndrome-2 (SARS-CoV-2), which were all caused by the members of the coronavirus family [1]. Beginning in November 2002, SARS was first identified at the end of February 2003 and quickly spread throughout the world [2]. More than 8000 SARS cases were reported, and the average mortality rate was 11% [2]. MERS was first reported in Saudi Arabia in September 2012 and 2,494 confirmed cases have been reported in 27 countries till now, with a total of 858 deaths [3, 4] Middle East coronavirus (MERS-CoV) may cause a viral respiratory disease, with symptoms of fever, cough, and shortness of breath. The 2019 novel coronavirus (2019-nCoV), which was later named SARS-CoV-2, was the third coronavirus resulting in a global public health crisis. COVID-19 first emerged at the end of the year 2019 in Wuhan city, Hubei province, China [5]. On 1 February 2020, the World Health Organization (WHO) proclaimed the epidemic as a global emergency [1]. Subsequently, the WHO declared the COVID-19 outbreak a global pandemic on 11 March 2020 [6]. At the time of 12:21 pm CEST, 4 April 2021, the number of global cases of COVID-19 has surpassed 130 million, including 2,839,588 deaths at least [7], more than those killed by SARS and MERS combined.

Since the outbreak of the disease, a large body of research on SARS-CoV-2 has been done. Estimation of dynamic features has been published under various assumptions to delineate hidden transmissions of the virus [8], [9], [10]. While estimates of those important quantities such as the reproduction number have been reported in the literature, those results are quite different and vary considerably across studies. The estimated reproductive number was also found differences across the regions, stages of infection, and the preventive measures applied [11, 12]. Different studies have been carried out on different populations under different conditions, and different authors may make various model assumptions. Since the epidemic began in China, and numerous papers have been published on this issue [9], only a few studies of systematic review and meta-analysis have focused on the basic reproduction number (R_0_) from a global perspective [10, 13], [14], [15]. In addition, some transmission routes of SARS-CoV-2 have not been clearly determined, and interpretation of the available findings must be coupled with the associated features of the studies.

To address all the issues mentioned above and update our knowledge of COVID-19, in the current review, we aim to provide an overview of COVID-19 transmission, and estimation of reproductive numbers based on worldwide’ literature. Moreover, the prevention measures against SARS-CoV-2, the development of vaccines, the vaccination threshold, and the equity issues of vaccination, have also been reviewed and discussed.

## SARS-CoV-2

COVID-19 and SARS have many similar features on transmission and pathogenicity. According to the studies on genome sequencing, SARS-CoV-2 is about 89% identical to bat SARS-like-CoVZXC21, 82% identical to human SARS-CoV [16]. However, the novel SARS-CoV-2 virus is spreading much more rapidly compared to the human SARS-CoV. This may be explained by the spike (S) proteins’ differences among coronaviruses [17, 18]. The S protein is one of the structural proteins encoded by the coronavirus genome, and both SARS-CoV-2 and SARS-CoV have the gene that expresses this protein. The spike structure on the virus surface is made by the trimers of S protein [19], and the S1 functional domain cleaved from the S protein is responsible for receptor binding. It is shown that the S protein of SARS-CoV-2 can bind with the same receptor of SARS-CoV to invade host cells, that is, angiotensin-converting enzyme 2 (ACE2) [20]. Xu et al. found that the binding free energy for SARS-CoV-2 S protein to bind with ACE2 is higher compared to SARS-CoV S protein (−50.6 vs. −78.6 kcal per mol) because of the loss of hydrogen bond interactions by replacing Arg426 with Asn426 [21]. Wrapp et al. found that the affinity of binding between SARS-CoV-2 and ACE2 is about 10- to 20-fold higher than ACE2 binding to SARS-CoV [22]. Therefore, the stronger binding affinity between SARS-CoV-2 S protein and ACE2 than that between SARS-CoV S protein and ACE2 may partly explain the rapid spreading of COVID-19 [23].

The continual mutations of SARS-CoV-2 have made the situation even more complex. Korber et al. [24] found the S protein amino acid change G614 had become the most prevalent form, which was associated with higher infectiousness. There exist at least three clades characterized by geographic and genomic specificity [25], which represent the different abilities of human-to-human transmission. They found the major prevalent genome in North America is GH (mutations in Spike D614G and ORF3a Q57H), whereas the most frequent clades in Europe and South America are GR. It is shown that the mutations of SARS-CoV-2 would make the pandemic varies in areas and in time.

Long time pandemic status has provided conditions for significant virus mutations. As of 30 November, 2021, there were four variants of the virus being announced as variants of concern (VOC), which is clearly associated with increased transmissibility, severity and/or decreased immunity. The first VOC is B.1.351 labelled as Beta by WHO. It was first detected in South Africa in May 2020. It is 50% more transmissible than the original virus and its ability to evade some vaccines was ascertained [26]. The second VOC is Gamma which was discovered in Brazil in November 2020. It is said that Gamma is 1.7–2.4 times more transmissible than original [27]. Due to the much higher transmissibility, the VOC Gamma had grown rapidly since December 2020, and it was responsible for the deadly second wave of COVID-19 in Brazil [28]. Another VOC dominating in Europe and the US is Delta. Since it was first discovered in October 2020, it has spread more than 100 countries around the world within 10 months. Researchers described it as an “improved” version of the Alpha variant and it was showed that the people infected with this variant had viral loads as much as 1,260 times higher than people infected with wild-type virus [29]. Many countries have fallen back into the epidemic and India is still stuck in the quagmire of the Delta variant. Last but not the least, South Africa reported a new variant B.1.1.529 in 24 November, 2021. Then, WHO designated this variant a variant of concern and named Omicron on 26 November, 2021 [30]. Although its transmissibility and the impact on current vaccines’ effectiveness is unclear, the number of new cases has increased rapidly within recent weeks in South Africa, and it has been predominantly the Delta variant. Moreover, some researchers suggest that there may be an increased risk of reinfection with Omicron [31]. This means that the government needs to spend more time and money to fight against the pandemic, and the endpoint of COVID-19 will be far beyond our eyesight.

New variants of the virus not only caused the rebound and prolongation of the epidemic, but also brought anxiety and panic. In the war against COVID-19, we have defeated waves of epidemics, but the endless VOCs have also made us so passive. It is understandable that many countries have taken strict non-pharmaceutical interventions (NPIs) since WHO designating Omicron, although the evidence of this new variant on transmissibility, severity of disease and effectiveness of infection is unclear. Long-term pandemic in many countries have facilitated the mutation of the virus, so that vaccine updates can never keep up with the speed of virus mutation. All these situations point to a worrying future, and taking strict NPIs to clear the infected cases in all countries within a short period may be the only way out. The inaccuracy of testing tools also fuels anxiety and fear, reliable and effective diagnostic testing kits are critical and essential against COVID-19, therefore, it should be considered in designing new testing kits to tackle these mutations and help vaccine developments.

## Transmission routes of SARS-CoV-2

### Transmission from animal to human

Coronaviruses (CoVs) belong to the order Nidovirales, family Coronaviridae, subfamily Coronavirinae, which has been identified to circulate in wild mammals and humans [32, 33]. It is most likely that the novel coronavirus SARS-CoV-2 has originated from bats due to its close phylogenetic relationship with beta-genus lineage b bat SARS-CoV [34]. Before spillover and adaptation to humans, coronaviruses most likely spread to intermediate hosts or animal models [34]. Subsequent to this, these intermediate hosts are increasingly vulnerable to co-infection of these coronaviruses, which also increases the chances of reassortment of viruses that lead to the emergence of novel genotypes, SARS-CoV-2 [35, 36]. Consequently, involving some enabling conditions, the COVID-19 with the potential of cross-species transmission ‘jump’ to humans and cause an outbreak in the wild animal wet markets of Wuhan, China [37], where wild animals of all kinds including bats were on sale. Evidence of the process of SARS-CoV-2 spreading from bats to humans can be found in the study of Wan et al. [34]. Based on the rich knowledge about SARS-CoV and the newly released sequence of COVID-19, Wan et al. found that the sequence of COVID-19 receptor-binding domain (RBD) is similar to that of SARS-CoV, and receptor-binding motif (RBM) of SARS-CoV-2 directly contacts human angiotensin-converting enzyme 2 (ACE2), strongly suggesting that SARS-CoV uses ACE2 as its receptor. In addition, while phylogenetic analysis indicates a bat origin of SARS-CoV-2, SARS-CoV-2 also potentially recognizes ACE2 from a diversity of animal species, implicating these animal species as possible intermediate hosts or animal models for COVID-19 infections [34].

### Transmission from human to human

#### Airborne transmission

The COVID-19 transmission has been confirmed to occur during close person-to-person contact, via respiratory droplets (>5–10 µm in diameter) and fomites from coughing or sneezing, similar to the transmission modes of SARS and MERS [38]. A study recently performed by Lelieveld et al. [39] estimated the infection risk for different indoor environments such as an office, a classroom, choir practice, and a reception/party. It showed that aerosols from super infective subjects (the top 5–10% of subjects with a positive test) can effectively cause COVID-19 clusters (>10 infections) in indoor environments [39].

As a direct dispersion of the SARS-CoV-2, microscopic aerosol particles (<5–10 µm in diameter) consisting of the evaporated respiratory droplets can remain airborne for 3 h, and the airborne transportation distance can reach 7–8 m [40, 41]. Si et al. investigated the behavior and fate of respiratory droplets (0.1–4 µm in diameter) during coughs in a single-path respiratory tract model. 2 µm droplets have the highest exhalation fraction from the pulmonary alveoli. The highest velocity of the exhaled droplets can reach 50 m/s for individual droplets [42]. Microscopic aerosol particles can be inhaled and penetrate deeply into the alveolar region of the lungs, readily causing COVID-19 infections in the indoor environment [39].

Alternatively, SARS-CoV-2 may combine with ambient aerosols and enter the human body. There are two possible mechanisms of ambient aerosols on the transmission of COVID-19 [41]. Firstly, ambient aerosols can be the carrier of COVID-19 to enter the human body. Secondly, the ambient aerosol can stimulate the expression of the SARS-CoV-2 receptor to accelerate the infection efficiency [41].

#### Faecal-oral transmission

Multiple studies highlight the threat of potential faecal-oral viral transmission of COVID-19 [43, 44]. In a recent study by Zuo et al. [43], faecal samples of 15 hospitalized patients with COVID-19 were collected, and it was found that 7 of 15 patients had stool positivity for SARS-CoV-2 by RNA shotgun metagenomics sequencing. The faecal viral metagenome of three patients continued to display active viral infection signature up to 6 days after clearance of SARS-CoV-2 from respiratory samples [43]. In addition, faecal samples with a signature of high SARSCoV-2 infectivity harbored a higher abundance of opportunistic pathogens, but live viruses were not to be able to be titrated or isolated from the faeces due to the methodological limitation [43]. A retrospective cohort study collected 3,497 respiratory, stool, serum, and urine samples from 19 January 2020 to 20 March 2020, to evaluate viral loads at different stages of disease progression in patients infected with SARS-CoV-2 in Zhejiang province, China [44]. RNA was detected in the stool of 55 (59%) patients and the serum of 39 (41%) patients. The urine sample from one patient was positive for SARS-CoV-2 [44]. Xing et al. [45] collected three pediatric cases of COVID-19 reported from January 17, 2020 to February 23, 2020 for Real-time fluorescence reverse-transcriptase-polymerase-chain reaction (RT-PCR) to detect SARS-CoV-2 RNA in throat swabs and fecal specimens. Viral RNA remained positive in the stools of pediatric patients after clearance of SARS-CoV-2 in the respiratory tract [45]. Two children had fecal SARS-CoV-2 turned negative 20 days after throat swabs showing negative, while that of another child lagged for 8 days [45]. These results suggested the potential for the virus to be transmitted through fecal excretion as well. As some studies reported to have prolonged viral RNA in rectal faecal samples [45, 46] more evidence is clearly required to accurately reveal the infectivity and pathogenesis of faecal-oral transmission of SARS-CoV-2 [43, 47].

#### Vertical transmission

Vertical transmission is defined as the transmission of the infectious pathogen from the mother to the fetus during the antepartum and intrapartum periods, or to the neonate during the postpartum period via the placenta *in utero*, body fluid contact during childbirth, or through direct contact owing to breastfeeding after birth [48]. There is limited but increasing evidence of SARS-CoV-2 infection during pregnancy. Several papers report the detection of SARS-CoV-2 RNA in pregnant women who received a coronavirus disease 2019 diagnosis [49], [50], [51]. A systematic review and meta-analysis of such studies found that a pooled proportion of 3.2% of neonates from mothers with COVID-19 have a positive result for severe acute respiratory syndrome coronavirus 2 viral RNA test using a nasopharyngeal swab. Severe acute respiratory syndrome coronavirus 2 viral RNA testing was positive in 2.9% of neonatal cord blood samples, and 7.7% of placenta samples [48]. Another systematic review on the possible transmission of SARS-CoV-2 through breast milk and breastfeeding found that break milk samples from 44.0% of the mothers diagnosed with COVID-19 were reported to be positive for SARS-CoV-2 RNA via RT-PCR analysis [52]. But among the cases with viral RNA detected in breast milk samples, one healthy neonate was fed with a breast milk substitute, while the maternal breast milk samples had detectable viral RNA and thus, it is not possible to ascertain the risk of infection by exposure to breast milk [51]. Given that most neonates born to mothers diagnosed with COVID-19 by RT-PCR tests during their pregnancy were negative for viral infection, and the majority of included studies did not assess the presence of SARS-CoV-2 specific antibodies in neonates, breast milk, placenta, or other tissues and hence, the evidence of possible SARS-CoV-2 transmission through breast milk is still limited [52]. Studies are needed with longer follow-up periods that collect data on infant feeding practices and viral presence in breast milk.

### Foodborne transmission

Although there is no direct proof that SARS-CoV-2 can be transmitted through food consumption [53, 54], concerns have been raised about the link between COVID-19 and contaminated frozen foods [53, 55, 56]. Han et al. [55] reviewed the literature on the persistence of SARS-CoV-2 on food surfaces and possible contamination of foods via the “farm-to-table” lifecycle, including the “cold chain”, to provide risk assessments on the foodborne transmission of COVID-19 and other human respiratory pathogens during pandemics. There have been a number of clustered outbreaks reported in food processing facilities since the onset of COVID-19. During April–May 2020, the US Centers for Disease Control and Prevention (CDC) identified 16,233 cases of COVID-19, including 86 deaths, among the workers at 239 meat and poultry processing facilities located in 23 states [57]. Food transmission evidence has been disclosed in China in early July 2020 by the detection of SARS-CoV-2 on frozen foods, including their packaging materials and storage environments, with two re-emergent outbreaks linked to contaminated food sources [55]. Recent studies found that SARS-CoV-2 remained highly stable on both refrigerated (4 °C) and freezing (−20 °C and −80 °C) foods for 14–21 days [58, 59], and SARS-CoV-2 may be introduced by contaminated cold-chain food sources via food imports [53]. It is necessary to adopt precautionary control measures on food contamination such as enhanced hand and respiratory hygiene, frequent disinfection of high-touch surfaces, isolation of infected workers and their contacts, as well as enhanced screening protocols for international seafood trade [53], otherwise a systematic risk for international spread of SARS-CoV-2 may occur [55].

## Epidemiological dynamics of SARS-CoV-2

### Reproduction number (R_0_)

Since the COVID-19 pandemic, a large number of epidemiological and mathematical modeling studies have focused on its transmission characteristics. An important concept in infectious disease research, the basic reproduction number (R_0_), has also been a hotspot. R_0_ also called the basic reproduction ratio or rate or the basic reproductive rate is a fundamental epidemiologic metric used to assess the contagiousness or transmissibility of infectious agents. The calculation of R_0_ is essential for implementing preventive measures as well [60, 61]. This index describes the expected number of infections generated by 1 case in a susceptible population. Typically, If R_0_ <1, the disease is controlled or not spreading too quickly. If R_0_ is 1, then 1 person is capable of spreading to 1 other person on average. If the R_0_ varies between >1, the disease can spread to a wider population (exponentially) from one single person, thus potentially creating an epidemic or pandemic [62]. To determine how deadly the COVID-19 is, it is fundamental to evaluate the R_0_ which was frequently referenced during the early stages of the disease [63].

R_0_ is not constant for a pathogen or a rate over time, and R_0_ cannot be modified through vaccination campaigns [64]. Although R_0_ is a biological reality, measuring it directly is far from straightforward. Because there are insufficient data collection systems in place to count the number of cases of infection during the early stages of an outbreak to help measure R_0_ most accurately [61]. For instance, in the early stage of SARS-CoV-2, insufficient test kits do not allow every potential patient with COVID-19-like symptoms to be tested, and there has been a good portion of asymptomatic COVID-19 carriers who would never be tested and counted as confirmed cases. As a result, R_0_ is nearly always estimated retrospectively from seroepidemiologic data with complex mathematical models developed using various sets of assumptions [65]. The estimation and interpretation of R_0_ are largely reliant upon understanding contact structure, the estimation method, and model inputs, and epidemiological parameters such as the infectious period and incubation period, which make R_0_ easily misrepresented [61], misinterpreted, and misapplied. Some R_0_ values reported in the scientific literature are likely obsolete. For example, For instance, in a systematic review reporting that the overall R_0_ was 3.32 (95% CI: 2.81–3.82), we found control reproduction (R_c_) numbers or time-varying effective reproduction (R_t_) numbers were wrongly extracted from several studies as R_0_ [66–69]. In another review concluding that the estimated mean R_0_ for COVID-19 is around 3.28, with a median of 2.79 and IQR of 1.16 [60], an R_t_ value was mistaken for R_0_ [70]. It is crucial to understand and utilize these metrics cautiously.

### A meta-analysis on the R_0_ estimation for COVID-19

#### Search strategy and selection criteria

This systematic review and meta-analysis were conducted to estimate the pooled R_0_ of COVID-19 in different counties via articles published in international journals. We searched PubMed, Web of Science, bioRxiv, and arXiv to obtain studies published between 1 December 2019 and 21 March 2021 regarding the reproduction number of COVID-19. We used the search terms or combinations of terms as the following – a) “Coronavirus Disease 2019” OR “COVID-19” OR “SARS-CoV-2”, b) “model” OR “modeling”, c) “reproductive number”. Studies were included if they presented a mathematical/statistical model of SARS-CoV-2 and reported R_0_ and at least one of the following parameters—R_t_, incubation period, generation time, latent period, or serial interval. Studies were excluded if they met the following conditions: a) Studies had conceptual confusion surrounding R_0_ and R_t_, b) Studies did not report necessary parameters like R_0_ with its 95% confidence interval (CI), c) Studies with R_t_ did not definitely discuss the interventions or the implementing period of the intervention was not reported, d) Simulation studies on R_0_/R_t_, e) Reviews comments, reports, and letters, f) Articles not in English. Three reviewers (the second author CSR, the third author JX and the fourth author WY) independently performed the literature search and screened titles and abstracts to exclude studies that did not meet our selection criteria before the full review of the full text. Disagreements were resolved by discussion among all authors.

#### Data extraction and statistical analysis

We used a predefined standardized extraction table to extract data. The extracted variables included the name of the first author, area of the study, period of data, model type and settings, estimated R_0_ (with a 95% confidence interval, CI), estimated R_t_/R_e_/R_c_ (with a 95% confidence interval, CI), and public health measures in countries if available. Data extraction was performed by CSR, JX and WY independently, and results were summarized by the co-first author ZYQ and WGH. Synthesis was carried out to aggregate the information from multiple studies with the same estimates (or effect size of interest) yet different features including the differences in the data collection, the sample size, and the conditions. A meta-analysis was conducted to obtain overall R_0_ and continental R_0_ values.

Heterogeneity between studies was assessed using the Cochran Q test, the I^2^ index, and T^2^ index (Table 1). According to the I^2^ results, heterogeneity can classified into the following three categories: I^2^<25% (low heterogeneity), I^2^=25–75% (average heterogeneity), and I^2^>75% (high heterogeneity) [71]. Because of the high I^2^ values that were calculated (99.1% for the globe, 99.4% for Asia, 98.4% for Europe, 90.9% for Africa, 97.6% for South America, and 84.1% for North America), as well as the significance of the Cochran Q test (p<0.0001), a random-effects model was used to estimate R_0_ in this work. Publication bias was assessed using Begg and Egger tests, as well as the funnel plots [72]. The bias was adjusted with trim and fill method [73]. Moreover, leave-one-out sensitivity analysis was also employed to assess the robustness of the results. Data were analyzed using R version 3.6.2 [74].

**Table 1:** Heterogeneity tests and pooled estimation of the basic reproduction number (R_0_) of COVID-19 by region.

Region	*Q*	*I* ^*2*^	*T* ^*2*^	Pooled estimate	95% CI
Globe	<0.0001	99.13%	0.4415	2.88	(2.70–3.06)
Asia	<0.0001	99.41%	0.2866	2.39	(2.08–2.70)
Europe	<0.0001	98.43%	0.8076	3.15	(2.86–3.45)
Africa	<0.0001	90.91%	0.4302	3.06	(2.27–3.86)
South America	<0.0001	97.63%	0.7974	2.86	(1.77–3.95)
North America	<0.0001	84.06%	0.4288	2.39	(1.40–3.38)

CI, confidence interval.

#### Results of COVID-19 R_0_ estimates

**Figure 1:**
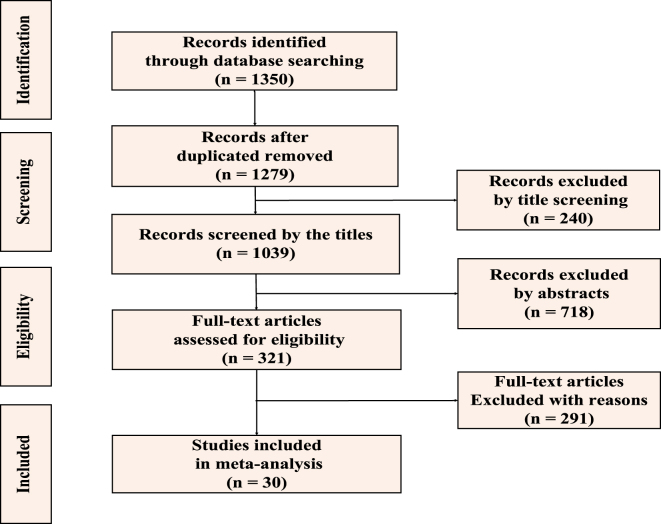
Flowchart of reports selection for inclusion in the systematic review.

**Figure 2:**
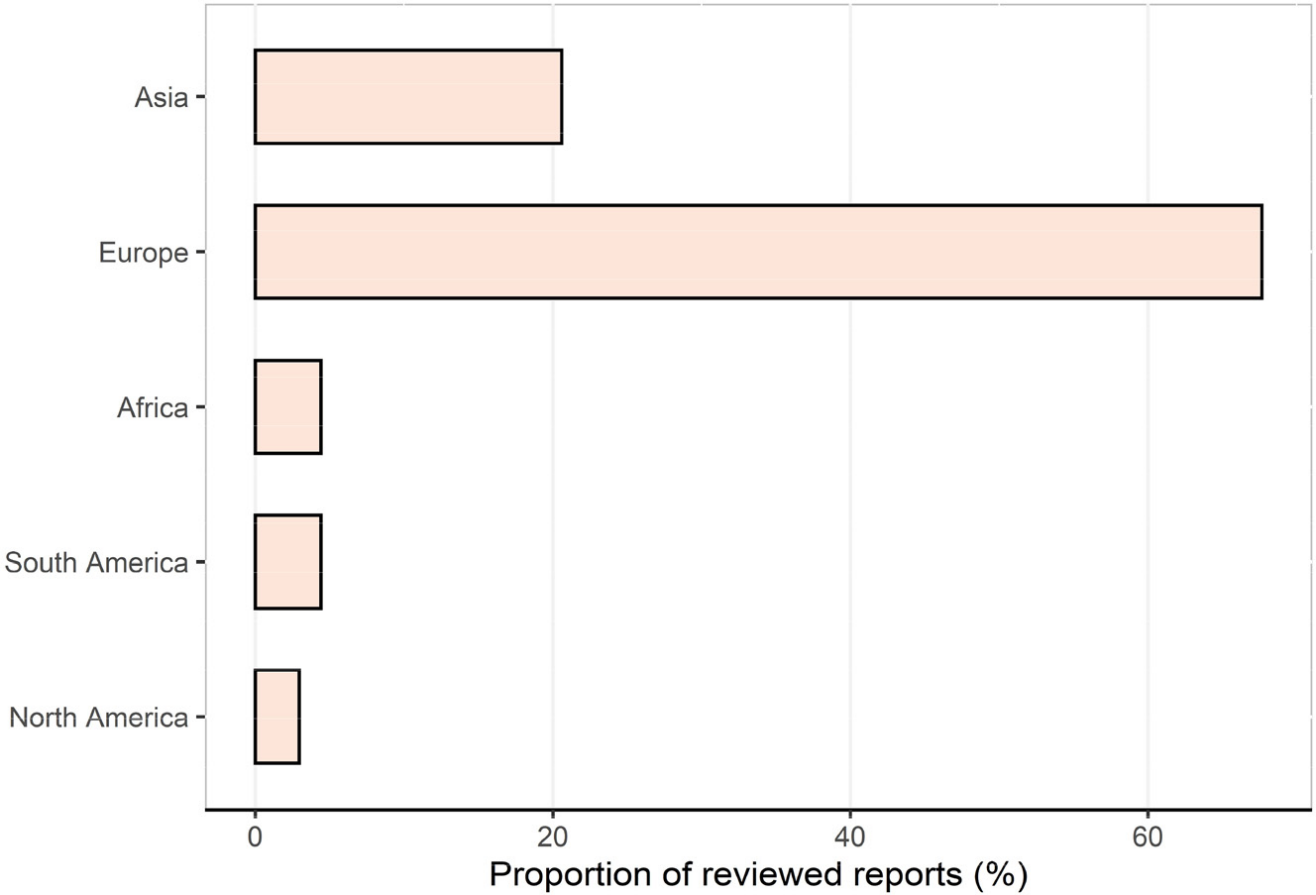
The proportion of records reporting R_0_ values in five continents. R_0_, the basic reproduction number.

We identified 1,350 studies, of which 71 were duplicates, which left us with 1,279 reports. A total of 1,039 reports passed the initial title screening, and 718 records were excluded by abstract, leaving 321 unique studies. Of these, 291 were excluded because they were simulation studies or did not report necessary parameters like R_0_ with its 95% confidence interval (CI). 30 publications passed the full-text assessment for eligibility (Figure 1). The 30 included studies with a total of 68 R_0_ records used a broad range of methods to generate R_0_. 63.3% (19/30) were compartment models, among which 26.3% (5/19) and 57.9% (11/19) of models belong to Susceptible-Infected-Recovered (SIR) models and Susceptible-Exposed-Infectious-Recovered (SEIR) models, respectively. 36.7% (11/30) of reviewed papers used other kinds of models such as the networked dynamic metapopulation model [75], the generalized growth model (GGM) [76, 77], the extended and modified Flaxman model [78], etc. 30 studies included in the meta-analysis reported estimated R_0_ and its 95% CI (Table 2). All the reports were conducted in 43 countries and regions in 2020, including much of East Asia, South Asia, and Europe, and a handful of countries in Africa and North and South America. The proportion of reviewed records in these five continents were shown in Figure 2.

**Table 2:** Descriptive characteristics of the records included in the meta-analysis.

Study	Study region	Study period	R_0_	LCL	UCL	Sources of cases
Adekunle et al. [79]	Nigeria	2020/02/27–2020/05/07	2.42	2.37	2.47	Confirmed cases
Rouabah et al. [80]	Algeria	2020/02/25–2020/05/24	3.78	3.03	4.53	Confirmed cases
Talmoudi et al. [81]	Tunisia	2020/02/29–2020/05/05	3.18	2.73	3.69	Reported cases
Chen Xu et al. [82]	Korea	Data collected about different countries were from the date when the first case in the country was reported to March 23, 2020	1.59	1.58	1.60	Confirmed cases
Genya Kobayashi et al. [83]	Japan	2020/03/1–2020/04/22	1.42	1.20	1.65	Confirmed cases
Hao Lei et al. [84]	China	2019/12/1–2020/03/11	2.12	2.02	2.21	Confirmed cases
Mohak Gupta et al. [85]	India	2020/03/04–2020/04/25	2.08	2.04	2.12	Confirmed cases
Muniz-Rodriguez et al. [76]	Iran	2020/02/19–2020/03/19	3.50	1.30	8.10	Reported cases
Muniz-Rodriguez et al. [76]	Iran	2020/02/19–2020/03/19	4.40	3.90	4.90	Reported cases
Quan-Hui Liu et al. [86]	China	2020/01/21–2020/04/19	2.40	1.60	3.70	Confirmed cases
Ruiyun Li et al. [75]	China	2020/01/10–2020/02/08	2.23	1.77	3.00	Reported cases
Sardar et al. [87]	India	2020/03/14–2020/05/03	2.03	1.66	2.33	Reported cases
Saurabh et al. [88]	India	2020/03/09–2020/07/06	1.62	1.07	2.17	Reported cases
Sugishita [89]	Japan	2020/1/14–2020/03/26	2.53	2.45	2.60	Reported cases
Toshikazu Kuniya [90]	Japan	2020/01/15–2020/06/30	2.60	2.40	2.80	Reported cases
Xingjie Hao et al. [91]	China	2019/12/08–2020/03/08	3.54	3.40	3.67	Confirmed cases
Zhang et al. [92]	China	2019/12/1–2020/4/21	2.33	1.96	3.69	Confirmed cases
Damjan Manevski et al. [78]	Slovenia	2020/03/04–2020/06/03	3.90	1.56	9.68	Confirmed cases
Jia Wangping et al. [93]	Italy	2020/01/22–2020/04/02	4.34	3.04	6.00	Confirmed cases
Kevin Linka et al. [94]	Austria	2020/05/10–2020/06/10	4.22	1.08	7.36	Confirmed cases
Kevin Linka et al. [94]	Belgium	2020/01/24–2020/06/10	4.30	3.59	5.01	Confirmed cases
Kevin Linka et al. [94]	Bulgaria	2020/01/24–2020/06/10	5.00	3.57	6.43	Confirmed cases
Kevin Linka et al. [94]	Croatia	2020/01/24–2020/06/10	1.29	1.21	1.37	Confirmed cases
Kevin Linka et al. [94]	Cyprus	2020/01/24–2020/06/10	0.93	0.50	1.36	Confirmed cases
Kevin Linka et al. [94]	Czech Republic	2020/01/24–2020/06/10	3.35	1.12	5.58	Confirmed cases
Kevin Linka et al. [94]	Denmark	2020/01/24–2020/06/10	2.92	2.00	3.84	Confirmed cases
Kevin Linka et al. [94]	Estonia	2020/01/24–2020/06/10	2.00	1.90	2.10	Confirmed cases
Kevin Linka et al. [94]	Finland	2020/01/24–2020/06/10	3.12	1.59	4.65	Confirmed cases
Kevin Linka et al. [94]	France	2020/01/24–2020/06/10	1.62	1.52	1.72	Confirmed cases
Kevin Linka et al. [94]	Germany	2020/01/24–2020/06/10	3.46	2.89	4.03	Confirmed cases
Kevin Linka et al. [94]	Greece	2020/01/24–2020/06/10	6.33	5.08	7.58	Confirmed cases
Kevin Linka et al. [94]	Hungary	2020/01/24–2020/06/10	1.66	1.42	1.90	Confirmed cases
Kevin Linka et al. [94]	Ireland	2020/01/24–2020/06/10	1.97	0.89	3.05	Confirmed cases
Kevin Linka et al. [94]	Italy	2020/01/24–2020/06/10	1.94	1.82	2.06	Confirmed cases
Kevin Linka et al. [94]	Latvia	2020/01/24–2020/06/10	4.25	3.43	5.07	Confirmed cases
Kevin Linka et al. [94]	Lithuania	2020/01/24–2020/06/10	2.50	0.76	4.24	Confirmed cases
Kevin Linka et al. [94]	Luxembourg	2020/01/24–2020/06/10	0.91	−0.81	2.63	Confirmed cases
Kevin Linka et al. [94]	Malta	2020/01/24–2020/06/10	2.42	0.05	4.79	Confirmed cases
Kevin Linka et al. [94]	Netherlands	2020/01/24–2020/06/10	2.08	1.81	2.35	Confirmed cases
Kevin Linka et al. [94]	Poland	2020/01/24–2020/06/10	5.88	4.16	7.60	Confirmed cases
Kevin Linka et al. [94]	Portugal	2020/01/24–2020/06/10	2.62	2.11	3.13	Confirmed cases
Kevin Linka et al. [94]	Romania	2020/01/24–2020/06/10	5.10	3.41	6.79	Confirmed cases
Kevin Linka et al. [94]	Slovakia	2020/01/24–2020/06/10	6.06	4.41	7.71	Confirmed cases
Kevin Linka et al. [94]	Slovenia	2020/01/24–2020/06/10	1.46	1.38	1.54	Confirmed cases
Kevin Linka et al. [94]	Spain	2020/01/24–2020/06/10	3.83	1.95	5.71	Confirmed cases
Kevin Linka et al. [94]	Sweden	2020/01/24–2020/06/10	5.19	4.21	6.17	Confirmed cases
Laura Di Domenico et al. [95]	France	2020/03/01–2020/04/05	3.18	3.09	3.24	Confirmed cases
Lemaitre Joseph et al. [96]	Switzerland	2020/02/24–2020/04/24	2.80	2.10	3.80	Confirmed cases
Mamon [97]	France	2020/03/19–2020/05/03	2.84	2.65	3.10	Confirmed cases
Mamon [97]	France	2020/03/19–2020/05/03	3.43	3.31	3.51	Confirmed cases
Seth Flaxman et al. [98]	Denmark	2020/02–2020/05/04	3.55	2.50	4.60	Confirmed cases
Seth Flaxman et al. [98]	Italy	2020/02–2020/05/04	3.39	3.03	3.75	Confirmed cases
Seth Flaxman et al. [98]	Germany	2020/02–2020/05/04	4.09	3.28	4.90	Confirmed cases
Seth Flaxman et al. [98]	Spain	2020/02–2020/05/04	4.76	3.98	5.55	Confirmed cases
Seth Flaxman et al. [98]	UK	2020/02–2020/05/04	3.82	3.30	4.34	Confirmed cases
Seth Flaxman et al. [98]	France	2020/02–2020/05/04	4.53	3.94	5.12	Confirmed cases
Seth Flaxman et al. [98]	Norway	2020/02–2020/05/04	3.04	2.16	3.92	Confirmed cases
Seth Flaxman et al. [98]	Belgium	2020/02–2020/05/04	5.16	3.88	6.45	Confirmed cases
Seth Flaxman et al. [98]	Austria	2020/02–2020/05/04	3.99	2.79	5.19	Confirmed cases
Seth Flaxman et al. [98]	Sweden	2020/02–2020/05/04	2.70	2.23	3.18	Confirmed cases
Seth Flaxman et al. [98]	Switzerland	2020/02–2020/05/04	3.26	2.60	3.93	Confirmed cases
Sypsa et al. [99]	Greece	2020/02/26–2020/04/26	2.38	2.01	2.80	Confirmed cases
Marissa L. Childs et al. [100]	USA	2020/02–2021/07	2.88	2.47	3.45	Reported cases
Worden et al. [101]	USA	2020/01–2020/05	1.87	1.38	2.62	Reported cases
Munayco et al. [77]	Peru	2020/02/29–2020/03/30	2.30	2.00	2.50	Confirmed cases
Pedro Alexandre da Cruz et al. [102]	Brazil	2020/02/25–2020/07/05	3.59	3.48	3.72	Confirmed cases
Tariq et al. [103]	Chile	2020/03/03–2020/11/02	2.58	0.78	3.99	Confirmed cases
Francisco Arroyo-Marioli et al. [104]	Part of the world	2020/01/23–2020/05/06	2.66	1.98	3.38	Reported cases

R_0_, basic reproductive number; LCL, lower control limit; UCL, upper control limit; SE, standard error.

The median R_0_ was 2.9 (IQR, 2.1–3.8) for all the reviewed studies. After stratified by continents, median R_0_ for Asia, Europe, Africa, South America, and North America over the whole outbreak periods were 2.4 (IQR, 2.1–2.6), 3.3 (IQR, 2.4–4.3), 3.2 (IQR, 2.8–3.5), 2.6 (IQR, 2.4–3.1) and 2.4 (IQR, 2.1–2.6), respectively. For publication bias assessment, the funnel plot (see Figure S1, Supplementary material), Begg test (p<0.0001), and Egger test (p=0.0241) suggested significant bias. The sensitivity analysis showed that no individual study significantly affected the pooled results of R_0_ (see Table S1, Supplementary material, which illustrates the sensitivity analysis results of meta-analysis for R_0_ estimates). According to the results of the random-effects model, the pooled R_0_ for COVID-19 was estimated as 2.9 (95% CI: 2.7–3.1), which means that each person infected with COVID-19 transmitted the infection to between 2 and three susceptible people on average. After stratified by continents, pooled R_0_ estimates for Asia, Europe, Africa, South America and North America were 2.4 (95% CI: 2.1–2.7), 3.2 (95% CI: 2.9–3.5), 3.1 (95% CI: 2.3–3.9), 2.9 (95% CI: 1.8–4.0) and 2.4 (95% CI: 1.4–3.4), respectively (Table 1). We stratified the analysis by sources of cases to make the R_0_ values comparable as well. Confirmed cases are people that have been tested, and the test confirms they have COVID-19 (i.e., a positive test). While the reported case figures on a given date do not necessarily show the number of new cases on that day – this is due to delays in reporting. The actual number of cases is likely to be much higher than the number of confirmed cases – this is due to limited testing. Confirmed cases have been used for R_0_ estimates 20 out of the 30 studies (Table 2). As demonstrated in Figure 3A and B, the pooled R_0_ estimate for confirmed cases (R_0_, 2.9; 95% CI: 2.7–3.1) was higher than that for reported cases (R_0_, 2.6; 95% CI: 2.3–2.9).

**Figure 3a:**
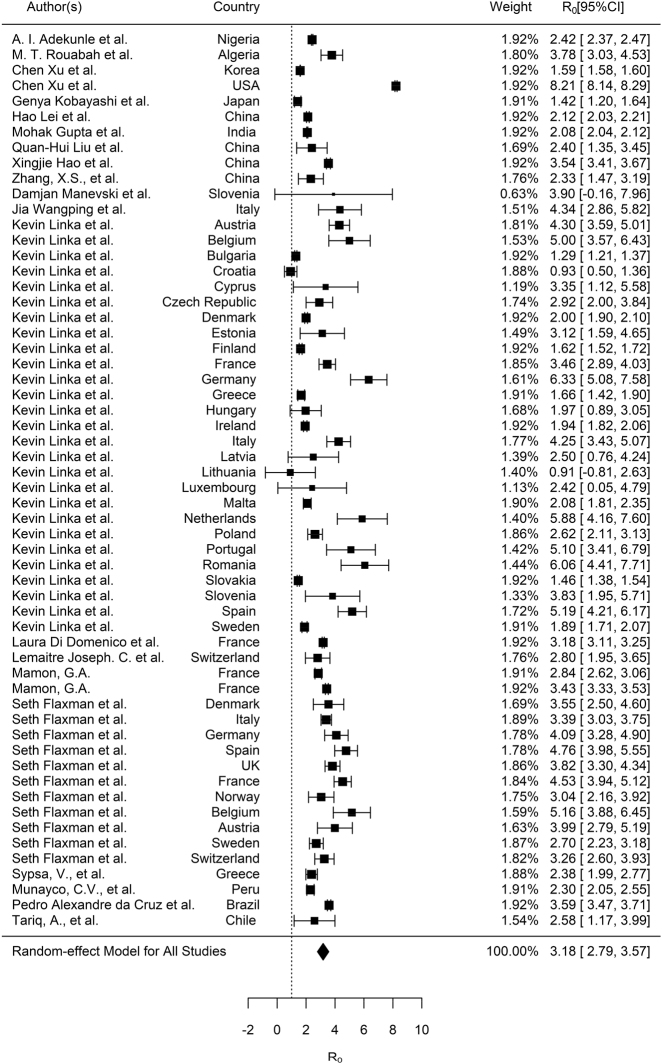
Meta-analysis of the synthetic estimated R_0_ for COVID-19 with confirmed cases. R_0_, the basic reproduction number. CI, confidence interval.

**Figure 3b:**
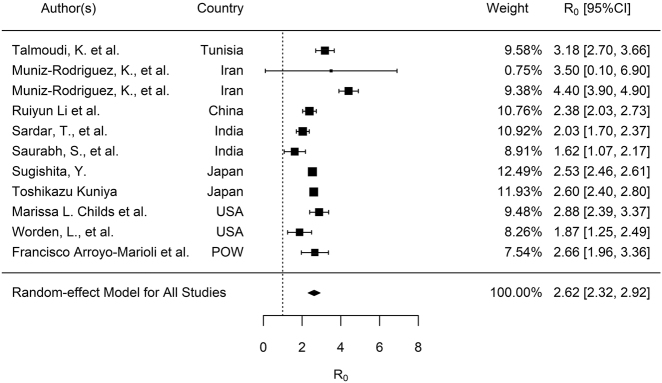
Meta-analysis of the synthetic estimated R_0_ for COVID-19 with reported case data. R_0_, the basic reproduction number. CI, confidence interval.

It is necessary to estimate the R_0_ of COVID-19 to determine the capability of transmission per primary infected person to the secondarily infected persons [105], as well as to provide a reference for the government to make interventions to control the spread of the disease [106]. Our systematic review and meta-analysis found that the overall R_0_ was 2.9 (95% CI: 2.7–3.1), which is similar to the results of an earlier review of 30 articles that were based on global level evidence (2.7) [10]. However, this estimate is lower than the previously summarized R_0_ values for the COVID-19 (3.15 [13] and 3.32 [9]). Our estimation is similar to the R_0_ values estimated for the MERS (R_0_<1–3) [107, 108], and is higher than R_0_ estimates for SARS (R_0_=0.58–1.17) [109]. Since the number of daily reported cases does not necessarily reflect the number of new cases on that specific date, which is because of the long reporting chain that exists between a new case and its inclusion in national or international statistics, R_0_ estimates were stratified by sources of cases. Our results showed that using reported cases in the early stage of epidemic can underestimate the R_0_. In our subgroup synthetic analysis, R_0_ values vary among different continents, that is R_0_ estimates for Asia, Europe, Africa, South America, and North America varied from 2.4 to 3.1. It is noted that the total value of R_0_ in a large population is the average of the R_0_ subtypes, indicating R_0_ is not an intrinsic characteristic of a given pathogen but rather describes the transmissibility of that pathogen within a specific population and setting [9]. In the most basic formulations of R_0_, its value fundamentally depends on three primary parameters: the duration of contagiousness from the infection onset, the probability of infection for each contact between a susceptible person and an infected person, and the contact rate [110]. In other words, R_0_ can be viewed as a combination of the pathogen’s biological constants and factors potentially influencing the contact rate, such as population density, social organization, and other epidemiological triads [61]. Because of the variation of social and human behavior among different areas, the transmission rate (β) and the recovery rate (γ) in epidemiological models are typically various. For example, Al-Raeei used eight countries’ reported COVID-19 data to fit the same SIRD (susceptible-infectious-recovered-death) model and found the coefficient of recovery range from 0.0008 in China to 0.0981 in India [111]. In addition to the socio-behavioral factors making the R_0_ different in areas, the regional discrepancy of COVID-19 mutations may also produce different R_0_. Mercatelli et al. suggested that a significant variation existed in the distributions of COVID-19 clades in different areas around the world [25]. Viruses mutations are of considerable medical and biological relevance as it has a great impact not only in the model estimation but also in the prevention and diagnosis of infectious disease. For instance, the early analyses announced by UK Prime Minister showed that a new strain of COVID-19 could increase the reproduction number by 0.4 or more [112]. Recently, the G614 mutation in the spike protein gene (D614G) has been calling scientific attention [113]. It was recently reported that this variant exhibits more efficient infection, replication, and competitive fitness in human airway epithelial cells, but maintains similar morphology and *in vitro* neutralization properties, compared with the ancestral wild-type SARS-CoV-2 virus [114]. A recent study found the potential link between viral genomic variation and its impact on transmission. The G614 mutation increases the rate of transmission of SARS-CoV-2, and hence the R_0_ [115]. Thus, the mutation distribution of COVID-19 in different areas should also be accounted for in the variation of estimated R_0_ if there are changes in the transmissibility of strains.

R_0_ estimates may be somewhat error-prone for reasons such as data insufficiency and the short period analyzed in the early stage of COVID-19. As more studies are done and more data are produced, the hope is that this error will be reduced. Thus modeling structures and assumptions may be another reason for the discrepancy of R_0_ across continents. The R_0_ values can be estimated through a variety of models, the three most popular methods were the Stochastic dynamic model-based method, Poisson likelihood-based (ML) method, and Exponential growth rate-based (EGR) method [116, 117]. Very few confirmed cases were reported in the early stage of the COVID-19 epidemic, which made high-quality data rarely available for mathematical models. The accuracy of R_0_ values mainly depended on model structures and assumptions about demographic dynamics. For instance, Exponential Growth (EG) and maximum likelihood estimation (ML) were applied to estimate the R_0_ of 2019-nCoV using data of confirmed 2019-nCoV cases before January 23, 2020. The R_0_ values estimated through the two different models were 2.90 (95% CI: 2.32–3.63) and 2.92 (95% CI: 2.28–3.67), respectively [118]. Hao showed the R_0_ value was about 3.5 according to the MSEIR model and about 1.5 according to the MSIR model utilizing the COVID-19 data from January 21 to February 17, 2020 [119].

In addition, the estimation accuracy of R_0_ was sensitive to the epidemiological parameterizations of models, which can be a reason for different R_0_ estimates in the same countries as well. Mohammad et al. [8] demonstrated that COVID-19 R_0_ is sensitive to the applied mean generation time when they estimated R_0_ in Qom, Iran. Zhou et al. [105] showed the value of R_0_ for SARS-CoV-2 was most sensitive to the generation time, so the more accurate estimation of the generation time based on the accumulation of epidemiological survey data would further improve the quality of the estimation of R_0_. For other pathogens, R_0_ values can be sensitive to model parameters as well. It was shown that R_0_ for SARS was most sensitive to the transmission rate and the relative infectiousness after isolation in hospitals [109]. A study of Echinococcosis in Xinjiang, China found that the initial value S_H_(0) can influence the R_0_ most compared to S_D_ (0), I_D_ (0), and other initial conditions [120].

## Public health measures against COVID-19

### Time-dependent reproduction number (R_t_) and intervention evaluation

Different from R_0_, the time-dependent reproductive number (R_t_) or the effective reproductive number (R_e_) can be utilized to partly achieve an explanation of the time course of an epidemic. R_t_ is defined as the actual average number of secondary cases per primary case at calendar time t (for t>0), which shows time-dependent variation due to the decline in susceptible individuals (intrinsic factors) and the implementation of control measures (extrinsic factors). If R(t)<1, it suggests that the epidemic is in decline and may be regarded as being under control at time t, which make R_t_ be used to characterize transmissibility once a certain proportion of the population has been infected and is resistant (immune) [121]. As R_t_ is similar to R_0_ in that it is a population-averaged value, it can also be estimated via a broad range of models. While common misinterpretation and misconceptions about R_t_ and R_0_ have been addressed [61], some misunderstandings and confusion persist [62]. With the evolution of the pandemic, the R_t_ can reduce as a result of the implementation of active measures or utilization of vaccination, thus R_t_ can be used to measure the effectiveness of vaccination campaigns or other public health interventions [122].

### Varying approaches of containment measures against COVID-19

**Figure 4:**
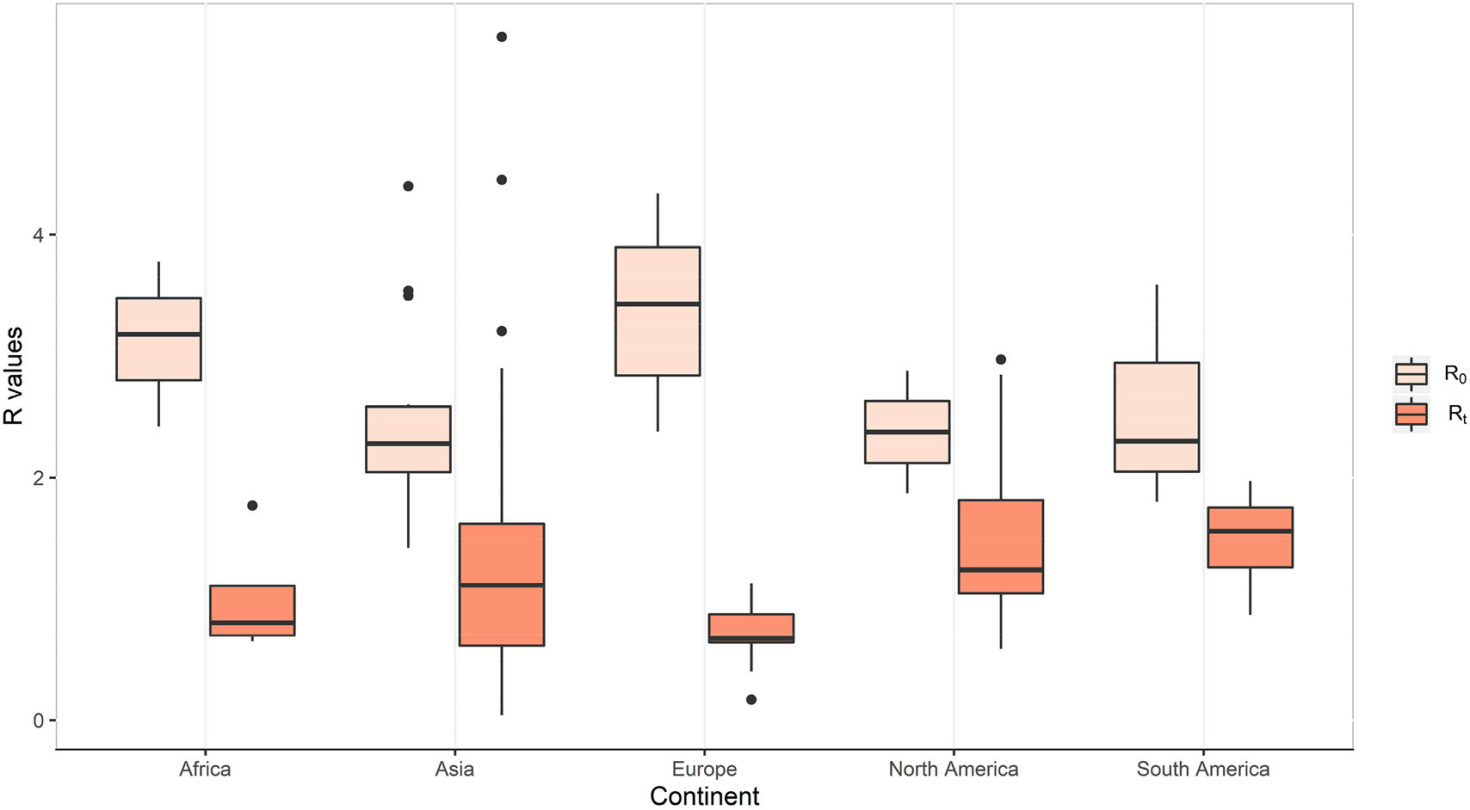
Boxplot of R_0_ and R_t_ metrics across continents. R_0_, the basic reproduction number. R_t_, the time-dependent reproduction number.

**Figure 5:**
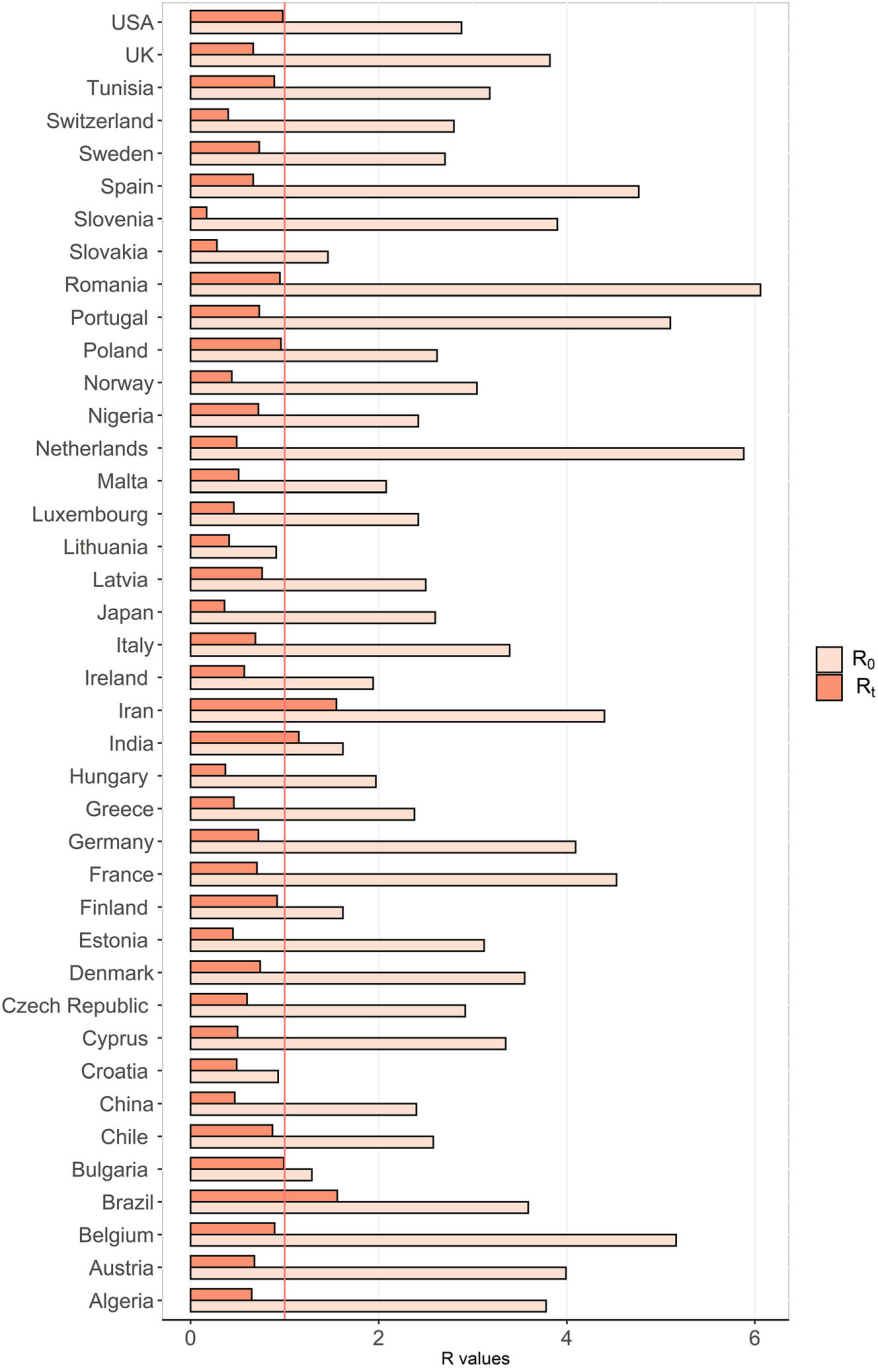
Comparison of R_0_ and R_t_ metrics across countries. R_0_, the basic reproduction number; R_t_, the time-dependent reproduction number.

To inform recommendations for control measures and determine the impact of varied intervention measures put in place, we reviewed R_t_ estimates in countries or regions across five continents. 1350 studies we identified were searched from several online databases as we describe in “Epidemiological dynamics of SARS-CoV-2” section. Studies were included if they presented a mathematical/statistical model of SARS-CoV-2 and reported R_0_/R_t_. Reports were excluded in this section if they met the criteria b), c), d), e) and f) mentioned in “Search strategy and selection criteria” section. The median R_t_ was 1.0 (IQR, 0.7–1.6) for all the reviewed studies. After being stratified by continents, Median R_t_ for Asia, Europe, Africa, South America, and North America were 1.1 (IQR, 0.6–1.6), 0.7 (IQR, 0.6–0.9), 0.8 (IQR, 0.7–1.1), 1.6 (IQR, 1.3–1.8) and 1.2 (IQR, 1.0–1.8), respectively. Estimated R_t_ values are lowest for Europe, followed by those for Africa, Asia, and North and South America. As demonstrated in Figure 4, the decrease of reproductive numbers varies across continents, which may imply the different effectiveness of containment measures taken by different countries. Thus, we extract studies reporting R_0_ with 95% CI and R_t_ with 95% CI to evaluate control measures in the 40 specific countries (see Figure 5).

#### Comprehensive interventions

Some countries took comprehensive control interventions against COVID-19. A study on the impact of interventions in limiting COVID-19 transmission in Sichuan, China estimated the number of cases averted by the implemented control strategies [86]. The outbreak resulted in 539 confirmed cases, lasted less than two months, and no further local transmission was detected after February 27. It may be attributed to a strict set of measures by the Sichuan government to deal with the outbreak, including case isolation, tracing and screening of contacts of confirmed cases, quarantine of travelers from affected areas, and screening of people’s temperature in public places [86]. According to a study by Lemaitre et al., Large-scale non-pharmaceutical interventions (NPIs) were implemented by the cantons and the federal government between 28 February and 20 March 2020 in Switzerland, including a set of measures: (1) ban on gatherings of more than 1,000 people, (2) school closure, (3) closure of non-essential activities, and (4) ban on gatherings of more than five people [96]. As a result, the national R_0_ was to be 2.8 (95% CI: 2.1–3.8) at the beginning of the epidemic. Starting from around 7 March, a strong reduction in reproductive number was found, with an 85.7% median decrease [96] (Table 3). Research suggests that the reproductive number of SARS-CoV-2 decreased by 95.6% since the adoption of NPIs, showing that NPIs were effective also in Slovenia for controlling SARS-CoV-2 (Table 3). R_t_ estimates of China, Japan, and 11 European countries were all below 0.5 (Figure 5), indicating that each sick person may infect fewer than 0.5 people on average, and the number of infected individuals may shrink over time. Generally, it will benefit a lot to implement early and continuing comprehensive interventions [123]. Nowadays, a large number of countries worldwide have conducted contact tracing to help identify infected people and contain the spread of COVID-19. In some countries, tracing “contact of a contact” has been used as well, which means any individual who is a contact of proximate contacts (i.e. spouse, children, co-workers, etc.) would be at risk and subject to quarantine once the proximate contacts had or developed symptoms, or tested positive for the virus causing COVID-19. This measure was always combined with modern technologies, such as cell phones, closed-circuit television (CCTV), and bank transactions, which helps collect information in an effective way, but privacy concerns and some ethical questions about exposing peoples’ activities and movements have been also raised [124].

**Table 3:** Summary of public health interventions against COVID-2019 in countries.

Country	Interventions	Type	Start date	End date	Implementing period, days	The reproductive number reduction, %
Algeria	Lockdown	Lockdown	2020/3/24	2020/5/24	61	82.80
Austria	Case based self-isolation mandated, Social distancing encouraged, Public events banned, School closure ordered, Lockdown ordered	Comprehensive interventions	2020/3/10	2020/5/4	55	83.01
Belgium	Case based self-isolation mandated, Social distancing encouraged, Public events banned, School closure ordered, Lockdown ordered	Comprehensive interventions	2020/3/10	2020/5/4	55	82.67
Brazil	Quarantine and social distancing	Isolation and social distance	2020/5/31	2020/7/5	35	56.50
Bulgaria	Public health interventions including isolation quarantine, physical distancing, and community containment.	Comprehensive interventions	2020/3/17	2020/5/10	54	23.26
Chile	Total lockdown	Lockdown	2020/5/5	2020/7/19	75	66.28
China	A set of measures; School closure; suspension of gathering activities and entertainment; case isolation, tracing and screening of contacts of confirmed cases, quarantine of travellers from affected areas, and screening of people’s temperature in public places.	Comprehensive interventions	2020/1/24	2020/4/19	86	80.40
Croatia	Public health interventions including isolation quarantine, physical distancing, and community containment.	Comprehensive interventions	2020/3/16	2020/5/10	55	47.31
Cyprus	Public health interventions including isolation quarantine, physical distancing, and community containment.	Comprehensive interventions	2020/3/10	2020/5/10	61	85.07
Czech Republic	Public health interventions including isolation quarantine, physical distancing, and community containment.	Comprehensive interventions	2020/3/10	2020/5/10	61	79.45
Denmark	Case based self-isolation mandated, Social distancing encouraged, Public events banned, School closure ordered, Lockdown ordered	Comprehensive interventions	2020/3/12	2020/5/4	53	79.18
Estonia	Public health interventions including isolation quarantine, physical distancing, and community containment.	Comprehensive interventions	2020/3/12	2020/5/10	59	85.70
Finland	Public health interventions including isolation quarantine, physical distancing, and community containment.	Comprehensive interventions	2020/3/12	2020/5/10	59	43.21
France	Case based self-isolation mandated, Social distancing encouraged, Public events banned, School closure ordered, Lockdown ordered	Comprehensive interventions	2020/3/13	2020/5/4	52	84.42
Germany	Case based self-isolation mandated, Social distancing encouraged, Public events banned, School closure ordered, Lockdown ordered	Comprehensive interventions	2020/3/6	2020/5/4	59	82.41
Greece	Nationwide lockdown	Lockdown	2020/3/23	2020/4/26	34	80.70
Hungary	Public health interventions including isolation quarantine, physical distancing, and community containment.	Comprehensive interventions	2020/3/11	2020/5/10	60	81.22
India	Home isolation of asymptomatic	Isolation and social distance	2020/5/10	2020/7/6	57	29.00
Iran	Social distancing	Isolation and social distance	2020/3/6	2020/3/19	13	64.80
Ireland	Public health interventions including isolation quarantine, physical distancing, and community containment.	Comprehensive interventions	2020/3/13	2020/5/10	58	70.62
Italy	Case based self-isolation mandated, Social distancing encouraged, Public events banned, School closure ordered, Lockdown ordered	Comprehensive interventions	2020/3/5	2020/5/4	60	79.69
Japan	Declaration of a state of emergency	Others	2020/4/7	2020/5/25	48	86.20
Latvia	Public health interventions including isolation quarantine, physical distancing, and community containment.	Comprehensive interventions	2020/3/13	2020/5/10	58	69.60
Lithuania	Public health interventions including isolation quarantine, physical distancing, and community containment.	Comprehensive interventions	2020/3/16	2020/5/10	55	85.70
Luxembourg	Public health interventions including isolation quarantine, physical distancing, and community containment.	Comprehensive interventions	2020/3/13	2020/5/10	58	80.99
Malta	Public health interventions including isolation quarantine, physical distancing, and community containment.	Comprehensive interventions	2020/3/13	2020/5/10	58	75.48
Netherlands	Public health interventions including isolation quarantine, physical distancing, and community containment.	Comprehensive interventions	2020/3/12	2020/5/10	59	91.67
Nigeria	International travel ban	Others	2020/3/20	2020/5/7	48	70.20
Norway	Case based self-isolation mandated, Social distancing encouraged, Public events banned, School closure ordered	Comprehensive interventions	2020/3/12	2020/5/4	53	85.70
Poland	Public health interventions including isolation quarantine, physical distancing, and community containment.	Comprehensive interventions	2020/3/12	2020/5/10	59	63.36
Portugal	Public health interventions including isolation quarantine, physical distancing, and community containment.	Comprehensive interventions	2020/3/12	2020/5/10	59	85.69
Romania	Public health interventions including isolation quarantine, physical distancing, and community containment.	Comprehensive interventions	2020/3/11	2020/5/10	60	84.32
Slovakia	Public health interventions including isolation quarantine, physical distancing, and community containment.	Comprehensive interventions	2020/3/12	2020/5/10	59	80.82
Slovenia	Non-pharmaceutical interventions (NPIs)	Comprehensive interventions	2020/3/10	2020/4/30	51	95.60
Spain	Case based self-isolation mandated, Social distancing encouraged, Public events banned, School closure ordered, Lockdown ordered	Comprehensive interventions	2020/3/9	2020/5/4	56	86.02
Sweden	Case based self-isolation mandated, Social distancing encouraged, Public events banned, School closure ordered, Lockdown ordered	Comprehensive interventions	2020/3/10	2020/5/4	55	73.01
Switzerland	Large-scale non-pharmaceutical interventions (NPIs): School closure; banned gatherings of more than five people and recommended voluntary home isolation for the whole population.	Comprehensive interventions	2020/2/28	2020/3/20	21	85.70
Tunisia	Lockdown	Lockdown	2020/3/22	2020/5/5	44	72.00
UK	Case based self-isolation mandated, Social distancing encouraged, Public events banned, School closure ordered, Lockdown ordered	Comprehensive interventions	2020/3/12	2020/5/4	53	82.58
USA	Shelter-in-place orders	Isolation and social distance	2020/3/17	2020/7/1	106	66.00

Decrease of the reproductive number (%)=(R_0_ − R_t_)/R_0_ × 100%.

#### Lockdown

Strict strategies of lockdown were imposed in some countries [80, 81, 99, 103]. For instance, a report of Chile on the effectiveness of lockdowns estimated that the reproductive number declined by 66.3% and reached 0.9 during lockdown (Table 3), indicating that the control measures at the start of the epidemic significantly slowed down the spread of the virus [103]. Large changes in R_t_ in response to the combined lockdown interventions were estimated for Tunisia as well [81]. The R_t_ moves to 0.89 (95% CI: 0.84–0.94) by the national lockdown measure, which is under the epidemic threshold 1 (Table 3). A nationwide lockdown restricting all nonessential movement throughout Greece began on March 23. A survey showed that before measures were implemented, the estimated R_0_ was 2.38 (95% CI 2.01–2.80) [99]. During the lockdown, daily contacts decreased by 86.9% and reproductive numbers decreased by 81.0% (Table 3). A total lockdown intervention applied in Algeria was effective in Algeria with a reduction of transmission by 82.8% [80] (Table 3). As shown in Figure 5, R_t_ estimates of Algeria, Greece, Tunisia, and Chile taking a control measure of lockdown were all below 1. Practically, a lockdown decision can have very extensive effects, it is also dependent on how prepared a country is in the early phases of the pandemic [125]. Moreover, the uneven enforcement of central and local authorities in each country has led to different outcomes. Simply lockdowns without adequate testing, targeted quarantines or other interventions will not be effective in the long run [126].

#### Isolation and social distance

Many countries adopted control measures of isolation and social distance. A study in Santa Clara County, California, the US estimated a shift to partial social distancing, combined with rigorous testing and isolation of symptomatic individuals, is a viable alternative to indefinitely maintaining shelter-in-place. It also estimated that if Santa Clara County had waited one week longer before issuing shelter-in-place orders, 95 additional people would have died by April 22 [100]. Similarly, a report in Iran estimated the reproduction number as 4.4 (95% CI: 3.9–4.9) by using a generalized growth model, and the reproduction number was 1.55 after social distancing interventions were implemented, with a decrease of 64.8% [76]. Literature shows that if social distancing measures are not respected, a difference of at least 100,000 cases may occur over the next 300 days in Brazil [102]. A report in Jodhpur, Rajasthan, in the northwestern part of India suggests that control measures of isolation can reduce transmission by 29.0% [88], which is lower than that of the USA, Iran, and Brazil (Table 3). It is noted that implementing period of home isolation in India was 57 days, almost two times the period of quarantine in Brazil, but the reduction of reproduction number in India was only half that of Brazil (Table 3). It may owe to the poor consciousness among the people. They may not take it seriously and go back to taking part in social functions without adhering to protocols and appropriate behavior. Strengthening propaganda and following the COVID-19 appropriate behavior continuously, the effectiveness of control measures will get better. In summary, compared with comprehensive interventions and lockdowns, taking only a measure of isolation likely is much less effective for SARS-CoV-2, which agrees with studies by Davies et al. [127] and Flaxman et al. [98]. It is noted that R_t_ estimates of Iran, Brazil, India and USA taking control measures of isolation and social distance were around 1 or over.

#### Other public health interventions

Other protection measures were taken as well. The Japanese government declared a state of emergency on April 7, 2020 since the serious growth of the daily number of newly reported cases started in late March [128]. The R_t_ during the period of the state of emergency was estimated to be 0.36, with a reduction in the reproductive number of 86.2% (Table 3), suggesting the state of emergency might have been highly effective on the first wave of COVID-19 in Japan [90]. A study in Nigeria implied that the measure of international travel ban put in place have not been effective enough, because it could only help reduce the relative infectiousness of imported cases, with a 70.2% decrease of transmission (Table 3). Efforts must be concentrated on ensuring that the existing measures are improved and additional measures better than the existing ones can be introduced [79].

## COVID-19 vaccines

### Strategies for COVID-19 vaccine development

The WHO has issued a target product profile that specifies the characteristics of an ideal vaccine [129]. And the US Food and Drug Administration has also issued a guidance document on COVID-19 vaccines [130]. Overall, both these documents require the ideal vaccine should have: a) an excellent safety profile suitable for multiple population groups, such as elderly, children, and pregnant women; b) no contraindications and minimal adverse events; c) at least 70% efficacy ideally within 2 weeks, and d) long-lasting protection that last for at least 1 year. Along with these guidelines, dozens of research teams have been involved in the development of the COVID-19 vaccine. From the experience of vaccine development for the previous MERS and SARS epidemic, we can summarize the strategies used by these research groups into six strategies, and each pipeline has its advantages and disadvantages (see Table 4) [131].

**Table 4:** Advantages and disadvantages of different COVID-19 vaccine pipelines.

Vaccine pipelines	Advantages	Disadvantages
Attenuated virus vaccine	Induce a strong and persistent immune memory	The attenuation of viruses is complex;
		Can be associated with significant side effects
Inactivated virus vaccine	Safer than live attenuated virus;	Long production time;
	Has development foundation	Immunity duration may be short
Viral vectored vaccine	Strong immune response;	Complicated manufacturing process;
	Mimicking natural infection	Immunity against the vector;
		Risk of genomic integration
Protein subunit vaccine	Safe and well-tolerated	Lower immunogenicity
DNA vaccine	Safe and well-tolerated;	Lower immunogenicity;
	Cold-chain free	Risk of genomic integration
RNA vaccine	Safe and well-tolerated	Instability;
		The potential risk of RNA-induced interferon response

#### Live attenuated virus vaccine

This is the most traditional technology used for vaccine development. It can provide the most immunogenic vaccine formations since the weakened microbes retain the ability to replicate *in vivo*. It has the ability to stimulate the immune system by inducing the toll-like receptors (TLRs) that involve B cells, CD4, and CD8 T cells [132]. However, this comes at the cost of safety issues, including the possibility of reversion to a virulent state and the risk of significant side effects [133]. Thereby, this kind of vaccine needs to be carefully assessed before proceeding to clinical use, and their storage and handling require strict procedures.

The WHO listed three preclinical ongoing studies focusing on live attenuated vaccine development [134]. The Serum Inst of India in collaboration with Codagenix Inc is developing a live attenuated vaccine based on their CodaVax technology. Accessible safety and immunogenicity of the Influenza vaccine in animal models were documented by using this technology [135]. The University of Hong Kong also starts from an influenza-based vaccine strain with a deletion in the NS1 gene (DelNS1-SARS-CoV2-RBD). They re-organized the gene to express the RBD domain of SARS-CoV-2 spike protein and cultivated it in the Madin Darby Canine Kidney Cells [132]. The University of Mehmet Ali Aydunar in Turkey and Indian Immunologicals Ltd in India also have projects on attenuated COVID-19 vaccines, while none of these vaccine candidates have stepped into phase three clinical trials.

#### Inactivated whole-virus vaccine

Vaccines that use inactivated microorganisms to induce immune response also have a very long history. Although it is a whole (killed) virus, it does not have the risk of viral reactivation compared with live attenuated viruses. Moreover, this vaccine is effective on multiple SARS-CoV-2 antigens, including the S protein. Therefore, the inactivated virus is stable and safe compared to live attenuated viruses [136]. A phase 2 clinical trial only reported mild side effects, such as localized injection site redness and pain, while all had resolved within 72 h [137]. There are strong research foundations of inactivated SARS-CoV-2 because this strategy has already been tested for SARS-CoV and many other diseases, such as Cholera, Hepatitis A virus, Plague, and the like. However, several limitations made inactivated whole-virus vaccines less attractive. First, it needs a long time to produce a dose of this kind of vaccine, so that it is not appealing enough in the time-sensitive race of COVID-19 vaccine development [138]. Besides, whole-inactivated vaccines have to be manufactured in biosafety level three-capable facilities, which will also cause difficulties for some areas [139]. Second, the duration of immunity is short that demands inoculation of higher amounts of vaccine with an adjuvant [136]. Third, the inactivation process may also structurally deforms the immunogenic epitopes of inactivated viruses which can disable the vaccine [131].

Although the inactivated whole-virus has some disadvantages, the proper combination of the inactivation method and adjuvants can also produce a satisfactory preventive effect. Sinopharm and Sinovac choose alum as the adjuvant [139, 140]. Among those inactivated candidates, the most promising inactivated COVID-19 vaccine candidates are in China [138]: CoronaVac/PiCoVacc (Registry index: NCT04456595NCT04582344), Inactivated COVID-19 vaccine (Vero cells) (Registry index: ChiCTR2000034780), and BBIBP-CoV (Registry index: NCT04560881 ChiCTR2000034780) have all stepped into phase 3 clinical trials.

#### Viral vectored vaccine

Viral vectored vaccines are another type of live attenuated vaccine. Viral vectors that cannot replicate in human cells are inserted specific DNA to induce a strong antigen-specific immune response. Although some replication active vectors can also be exploited as viral vector vaccines, they will not trigger any other disease. Ura et al. suggested that this kind of vaccine is highly specific in delivering the genes to the target cells, highly efficient in gene transduction, and induces immunity [132, 141]. Because researchers are only exposed to viruses and DNA that do not cause diseases, the risk of laboratory infection is greatly reduced. Some drawbacks also exist in the process of viral vectored vaccines production. Because we need to insert only DNA into viral vectors, including cellular system optimization and contaminants exclusion, complicated technologies are needed to ensure the efficiency of viral vectors [142]. Moreover, pre-existing immunity on the viral vector may affect vaccine efficacy. For instance, the most widely used vectors for COVID-19 vaccines are adenoviral-based. Most patients may have pre-existing immunity against the adenovirus, and the immune response to SARS-CoV-2 may be dampened [143]. Last, there is a risk of genomic integration of recombinant viruses into the host which may lead to cancer, and additional long-term biosafety assessment is required.

Kaur et al. [132] suggested that viral vector vaccines had great potential for prophylactic use. There are some candidates in phase 3 clinical trials. ChAdOx1 (NCT04516746NCT04540393) was developed by the University of Oxford, and the Adenovirus vector genome is formatted by inserting the SARS-CoV-2 spike gene into the E1 locus of the ChAdOx1 adenovirus genome. Ad5-nCoV (NCT04526990NCT04540419) developed by the Beijing Ins of Biotechnology was constructed by the Admax system from the Microbix Biosystem [144]. Both RBD and spike protein-specific neutralizing antibody increases were observed within two weeks of immunization, whereas the pre-existing anti-Ad5 immunity would limit the immune response [144]. Beyond these two projects, Gam-COVID-Vac (NCT04530396NCT04564716) and Ad26.COV2⋅S (NCT04505722) also have the potential of being marketed within a short time.

#### Protein subunit vaccine

This kind of vaccine consists of viral proteins or protein fragments. Because it does not have any live component of the viral particle, it is one of the safest candidates with fewer and milder side effects. For the biosafety for a new virus causing a global pandemic, numerous vaccine projects are focusing on the SARS-CoV-2 proteins and their fragments. As a cost of safety, the subunit vaccine can only induce limited immunity and requires auxiliary adjuvant to activate a robust immune response. Most of the SARS-CoV-2 subunit vaccines have focused on the spike protein or its fragments, such as the RBD [145]. Because of the activation of antibodies and contains HLA-restricted T-cell epitopes in the studies of MERS and SRAS-CoV, the N protein has also drawn some attention [146, 147].

NVX-CoV2373, produced by Novavax, is the leading candidate for the SARS-CoV-2 protein vaccine. This vaccine uses a saponin-based adjuvant to increment the adaptive immune responses to recombinant Ebola virus glycoprotein vaccines and MVA-based influenza vaccines [148]. This candidate uses a nanoparticle with the full-length spike protein that was capable of binding ACE2 receptors with high affinity. Instead of using the full-length spike protein, another candidate produced by Anhui Zhifei Longcom (NCT4464085) only utilizes the RBD of the spike protein. The University of Queensland in collaboration with GSK and Dynavax intends to use the Molecular Clamp technology to produce the neutralizing antibodies and use the adjuvant platform technology to enhance the vaccine response [132].

#### DNA vaccine

DNA-based vaccination approach is said to be the most revolutionary approach that encodes for the antigen [132]. DNA-based vaccination approach can increase the stability of the DNA molecule so that it can be used to construct a large number of mRNA molecules. Moreover, because the DNA vaccine is stable at room temperature, there is no need for a cold chain for this kind of vaccine. There are few DNA vaccines registered for human use, and the pipelines of DNA-based candidates for SRAS-CoV-2 can be divided into two ways: naked DNA plasmids and naked DNA plasmids plus electroporation [136].

INO-4800, produced by Inovio Pharmaceuticals, is a prophylactic DNA vaccine against COVID-19. The vaccine was created based on the synthesized SRAS-CoV-2 IgE-spike sequence, and it was suggested that the vaccine can provide an effective immunity within a week after vaccination [149]. In the USA, there are more than three registered DNA vaccine candidates (https://clinicaltrials.gov/), but no additional information is available.

#### RNA vaccine

Although messenger RNA (mRNA) has not yet produced any registered vaccine in the past, the production of nucleic acid-based vaccine is often faster and cheaper than protein subunit vaccines [150], so that there have been six RNA vaccines for COVID-19 in the phase of clinical trials [134]. This kind of vaccine consists of viral antigen-encoding mRNAs that can be translated by human cells to produce antigenic proteins and induce immunity. Similar to DNA vaccines, RNA vaccines are easy to modify the antigenic proteins, while they will not interact with host-cell DNA and thus no genomic integration risk exists. The biggest challenge of RNA vaccines is the propensity of mRNA to degrade. The vaccine is not as stable as the DNA vaccine and it must be stored and handled carefully. In addition, there is a potential risk of RNA-induced interferon response which is associated with inflammation and autoimmunity [151].

The mRNA-1273 vaccine produced by Moderna in collaboration with the National Ins of Allergy and Infectious Diseases is the leading RND vaccine candidate of SRAS-CoV-2. It encodes the spike-2 protein antigen and they substituted the amino acids at 986 and 987 with proline to stabilize the protein [131]. Several studies suggested that the mRNA-1273 vaccine is safe and effective in preventing COVID-19 [152, 153].

### Effectiveness of current vaccines

As of November 2021, seven vaccines have been approved for full or emergency use by WHO: Pfizer–BioNTech, Oxford–AstraZeneca, Sinopharm BIBP, Moderna, Janssen, CoronaVac and Covaxin. Table 5 showed the efficacy and effectiveness of these vaccines from recent clinical trials and real world evidence, respectively. RNA-based vaccines have the best protection effect and the efficacy of viral vectored vaccines is not higher than 80%. Due to the impact of variants, the effectiveness from real world studies all decreased compared to that from randomized clinical trials (RCT). It is showed that many variants of CoV can partially escape the immune barrier constructed by the vaccines. How different variants evade the vaccines can be seen in other place [154].

**Table 5:** Efficacy and effectiveness of COVID-19 vaccines approved for full or emergency use.

Vaccine	Type	Effectiveness for symptomatic infection	
		RCT	Real world
Pfizer–BioNTech	RNA based vaccine	91.3 (89.0–93.2) [155]	83.0 (78.0–87.0) [156]
Moderna	RNA based vaccine	93.2 (91.0–94.8) [157]	90.0 (68.0–97.0) [158]
Oxford–AstraZeneca	Viral vectored vaccine	74.0 (65.3–80.5) [159]	61.0 (51.0–70.0)
Janssen	Viral vectored vaccine	66.0 (55.0–75.0) [160]	NA^a^
Sinopharm BIBP	Inactivated virus vaccine	78.1 (64.8–86.3) [161]	NA^a^
CoronaVac	Inactivated virus vaccine	83.5 (65.4–92.1) [162]	65.9 (65.2–66.6)
Covaxin	Inactivated virus vaccine	77.8 (65.2–86.4) [163]	NA^a^

^a^NA: not available.

### The expected vaccination coverage of COVID-19

After a development racing of COVID-19 vaccines, many candidates have been approved around the world. For instance, there are five types of vaccines approved by the National Food and Drug Administration in China, three for inactivated vaccines, one for recombinant subunit vaccine, and one for adenovirus vectored vaccine. To build up herd immunity as soon as possible, governments have tried their best to encourage people to be vaccinated. However, not all countries can afford the cost of vaccination for the whole population. What coverage threshold should each country reach is an important question for decision making. In other words, how many people in the world or a country will need to get a COVID-19 vaccine?

From the point of mathematic, as the meaning of reproductive number (R), when each individual infects on average more than one, the scale of infected people will continue to grow exponentially; and when the R is less than one, the number of cases will gradually fall. Moreover, we can use R(t) to evaluate the effect of public health measures implemented by the government. On the other hand, we can also use R(t) to predict the extent of vaccines we at least use to control the pandemic and get herd immunity. If we refer to critical vaccine coverage r*, which is the proportion of randomly chosen individuals in the population that must be vaccinated to achieve R(t)<1. If we have a vaccine that reduces transmission by a factor *α* then we can get the expected vaccination coverage rate:

$$r^{*}=\frac{1}{\alpha }\left(1-\frac{1}{R_{t}}\right)$$

In some textbooks, the R(t) will be replaced by R_0_ to represent the “getting back to normal” without any other immunity or control measures.

Obviously, *α* and *R* are two important inputs, and the uncertainty of these numbers will dramatically influence the result. As we mentioned above, discrepancies in model specification, the scope of data, and even the population will make the reproductive number vary greatly. Therefore, we can use the pooled estimate of R_0_ from meta-analysis to reduce this uncertainty.

What’s more, the *α* varies across areas because different countries have chosen different vaccines. We calculated the critical coverages by assuming one vaccine used for the whole world in each case.

In the previous section, we get the pooled R_0_ in the world is 2.88 (95% CI: 2.70–3.06). If we use the minimum requirement of efficacy (70%) listed by WHO, there must be at least 93.3% (95% CI: 89.9%–96.2%) people being vaccinated. Specifically, the coverage rate in Asia needs to be 83.1% (95% CI: 74.1%–89.9%), while the expected coverage rate in Europe is the highest (97.5% [95% CI: 92.9%–100.0%]).

According to the most recent RCT results of vaccines, if only Modera’s vaccine were used, the coverage rate should range from 62.4% to 73.2%. However, the evidence from real-world suggested that the critical coverage should be 64.6–75.8%. This coverage means billions of dollars have to be spent and billions of people should be vaccinated. And the real efficacy of vaccines used in many countries was lower than 93.2%, which means a much higher cost. Moreover, we can see a great discrepancy in coverage rates for different continents in Table 6. No matter which effectiveness level, the coverage rates in Europe and Africa need to be 10% higher than in other regions.

**Table 6:** Expected coverage rate of Modera’s vaccine for different continents.

	WHO	RCT	Real-world
Asia	83.1 (74.1, 89.9)	62.4 (55.7, 67.6)	64.6 (57.7, 70.0)
Europe	97.5 (92.9, 100.0)	73.2 (69.8, 76.2)	75.8 (72.3, 78.9)
Africa	96.2 (79.9, 100.0)	72.2 (60.0, 79.5)	74.8 (62.1, 82.3)
South America	92.9 (62.1, 100.0)	69.8 (46.7, 80.1)	72.3 (48.3, 83.0)
North America	83.1 (40.8, 100.0)	62.4 (30.7, 75.6)	64.6 (31.7, 78.2)

It is a great challenge for all countries to produce such a large quantity of vaccines. A single country can’t provide all the vaccines required worldwide. But currently, most countries are still not able to meet the needs by themselves. There is a big problem of vaccine resources distribution, and this is why WHO urgently calls for the equity of the COVID-19 vaccine.

### Equity in COVID-19 vaccine distribution

#### Inequities among countries

Globally, to avoid the inequity in COVID-19 vaccine distribution among countries and to prevent a repeat of vaccine protectionism like the H1N1 scenario [86], the COVID-19 Vaccine Global Access (COVAX) Facility was established by WHO in April 2020 and was co-led by the Vaccine Alliance (Gavi), the Coalition for Epidemic Preparedness Innovations (CEPI). With lower prices for low-income and middle-income countries, COVAX aims to provide all countries the opportunity to get at least 20% population vaccinated at the end of 2021. Under its vision, no country can be the first to vaccinate over 20% of its population if other countries cannot reach this rate [164].

Unfortunately, COVAX has not achieved its goal yet. Although the global administered coverage of the second dose has reached 15.5% in 9th Aug 2021, the United Kingdom, France, and the USA have vaccinated for more than 40% of its population, while the rates in low- and middle-income countries like Zambia, Sudan, and Nigeria are even lower than 10% (https://ourworldindata.org/grapher/covid-vaccination-doses-per-capita). Because COVAX cannot make itself the only way to purchase vaccines, many high-income countries with smaller populations directly purchase vaccines from the developer instead of COVAX, which makes them host most of the vaccines in the world [165]. Moreover, insufficient funding has also prevented COVAX from providing an adequate share of vaccines to low- and middle-income countries that are more in need of them [166].

Besides the lack of a global vaccine stockpile, some researchers have also criticized the allocation strategies of COVAX [167, 168]. At the first stage in COVAX’s allocation structure, a proportional allocation strategy will be implemented for every country until all countries have reached the coverage goal. However, when high-income countries have broken the role on the distribution of vaccines, equity in population is no longer equal to fairness in need. Some additional allocation models based on ethical equity have been proposed as complements of COVAX, such as the fair priority model (FPM) [168] and multi-value ethical framework (MEF) [86].

#### Inequities among populations

When limited vaccines are available in the early stage of the vaccination program, policymakers have to decide on who should be vaccinated first. Accounting for both health and economic benefits, all countries face a multicriteria decision problem. As recommended by WHO, many countries have given priority to key workers, clinically vulnerable groups, and elderly groups under the structure of the Strategic Advisory Group of Experts on Immunization (SAGE) [169, 170]. However, pregnant women, an important vulnerable group, were excluded from the recruitment lists of most of the global clinical trials [171]. Without evidence about safety and efficacy during pregnancy, pregnant women have fallen into a perpetuated cycle of exclusion [172]. When pregnant women are not considered in early efforts of vaccine development, they are in turn excluded from generations of evidence, and they are excluded from vaccination programs. Ignoring pregnant women, who have equivalent or even worse outcomes compared with non-pregnant groups, are harmful to the global COVID-19 disease burden and are unjust in the progress of global immunization of COVID-19 vaccines.

Many researchers have advocated the inclusion of pregnant groups [172–174], and the Pregnancy Research Ethics for Vaccines, Epidemics, and New Technologies (PREVENT) Working Group even issued 22 recommendations to promote equity for pregnant women in vaccine development and response [175]. Although pregnant women were not in most of the cohorts of trials, some pregnancies occurred across the trials of three vaccines previously approved in the UK [176]. The data showed no significant detrimental effect of vaccination on pregnancy. Thus, the United Kingdom, the United States, and European Union all recommended pregnant people to be vaccinated if the benefits outweigh the potential risks. Moreover, Shimabukuro et al. [177] provide more scientific evidence about vaccine safety in pregnant persons. Based on a vaccination health checker surveillance system data of 35,691 participants in the USA, they find no obvious safety signals among pregnant persons who received mRNA COVID-19 vaccines. Although it is said that more longitudinal follow-ups with large numbers of women vaccinated earlier in pregnancy are needed, at least the mRNA vaccines of COVID-19 are most likely adequate for the pregnant group. As mentioned in the previous section, mRNA vaccines are not very stable and have high requirements for the cold chain. Due to the high cost of cold chain transportation, there is no well-operated cold chain network in many low-income countries. Even if mRNA vaccines are completely safe and effective for pregnant women, the inequity of pregnant women vaccination still exists in low-income countries.

Racial inequities in vaccination also persist. Take the United States as an example, from the latest data on COVID-19 vaccinations by race, nearly two-thirds of people who had received at least one dose of the vaccine were White (58%), and only 10% were Black and 6% for Asian [178]. Black people have only received a small share of vaccination, whereas more than half of cases and deaths were made up of them. Despite the hesitancy for the vaccine, a long time of medical racism and misinformation has caused the unfairness of vaccination for Black [179]. CDC in the USA has made many efforts to ensure that Black and other minorities have fair and just access to COVID-19 vaccination. But as the mantra of the COVID-19 pandemic, “No one is safe until everyone is safe” [180], the efforts of filling the gap in the vaccination of minorities are far from enough.

## Conclusion

COVID-19 has raised serious concerns globally. As of April 4, 2021, at least 130 million cases and 2 million deaths associated with SARS-Cov-2 have been recorded worldwide. Confirmed to occur during close person-to-person contact, the major transmission route of COVID-19 is airborne transmission. Aerosol particles consisting of respiratory droplets can be a direct dispersion of the SARS-CoV-2, and ambient aerosols can be the indirect ones. Thus it is necessary to implement social distancing interventions and reduce aerosol pollution levels to bring this epidemic under control. There is no determined proof for faecal-oral, vertical, and foodborne transmission, more evidence is required to accurately reveal the infectivity and pathogenesis of these transmission routes of SARS-CoV-2. Curbing COVID-19 infections primarily demands efforts from every country. It is noted that in the absence of drastic action, even the best health systems can be inefficient. Comprehensive interventions (suspension of gathering activities and entertainment, case isolation, tracing and screening of contacts of confirmed cases, and screening of people’s temperature in public places) and lockdowns would be effective for reducing COVID-19 transmission and avoiding future outbreaks of SARS-CoV-2. It is necessary for all the countries to rethink the use of more tailored measures if COVID-19 is to be with us for years to come. Besides, countries and regions worldwide must strengthen coordination and cooperation to control the epidemic.

The global effort to develop safe and effective COVID-19 vaccines has yielded remarkable results. In the race of COVID-19 vaccine development, six strategies were implemented to create effective candidates to control the SARS-CoV-2 replication at the individual level. Many of these candidates were based on the spike protein or its subdomain of the SARS-CoV-2, whereas variants of CoV can partially evade current vaccines. Thus, the critical coverage rate would be higher and the development of new vaccines is always on the road. The vaccination program of COVID-19 is also not for a single country or area. Vaccination to achieve control of the pandemic with no public health measures still requires huge resources, even if the best vaccine candidate is used for everyone. Great equity problems exist in the distribution of vaccines among countries and people groups. Although we have made some efforts to provide low- and middle-income countries and vulnerable groups more opportunities of vaccination, we need to carry out more international cooperation in COVID-19 vaccine research and development so that each country can afford the burden of vaccination and balance the shares of vaccines among groups in its own country. Before herd immunity is reached to block mutations, the use of comprehensive interventions and vaccines together is essential for the most effective control of COVID-19.

## Supplementary Material

Supplementary Material Details
