# Supplementary material for: A review on COVID-19 transmission, epidemiological features, prevention and vaccination
Source: Med Rev (2021). 2022 Mar 2;2(1):23–49. doi: 10.1515/mr-2021-0023 (PMC9047653; doi:10.1515/mr-2021-0023)
Supplement: Supplementary file 1 — Supplementary Material Details [file j_mr-2021-0023_suppl_001.docx]

*Supplementary Material*

**A review on COVID-19 transmission, epidemiological features, prevention and vaccination**

**Yuqin Zhang ^#1^, Gonghua Wu ^#1^, Shirui Chen ^1^, Xu Ju ^1^, Wumitijiang Yimaer ^1^, Wangjian Zhang ^1^, Shao Lin^2^, Yuantao Hao ^*1,3^ , Jing Gu ^*1^, Jinghua Li ^*1^**

1. School of Public Health, Sun Yat-Sen University, Guangzhou, 510080 China

2. Department of Environmental Health Sciences, School of public health, University at Albany, State University of New York, Rensselaer, New York, 12144 USA

3. Sun Yat-Sen University Global Health Institute, School of Public Health and Institute of State Governance, Sun Yat-Sen University, Guangzhou, 510080 China

*Correspondence to: [haoyt@mail.sysu.edu.cn](mailto:haoyt@mail.sysu.edu.cn); [gujing5@mail.sysu.edu.cn](mailto:gujing5@mail.sysu.edu.cn); [lijinghua3@mail.sysu.edu.cn](mailto:lijinghua3@mail.sysu.edu.cn)

**^#^**Yuqin Zhang and Gonghua Wu contributed equally to this work

NUMBER OF TABLES: 1

NUMBER OF FIGURES: 1

**Figure S1 Funnel plot for mata-analysis of R_0_**


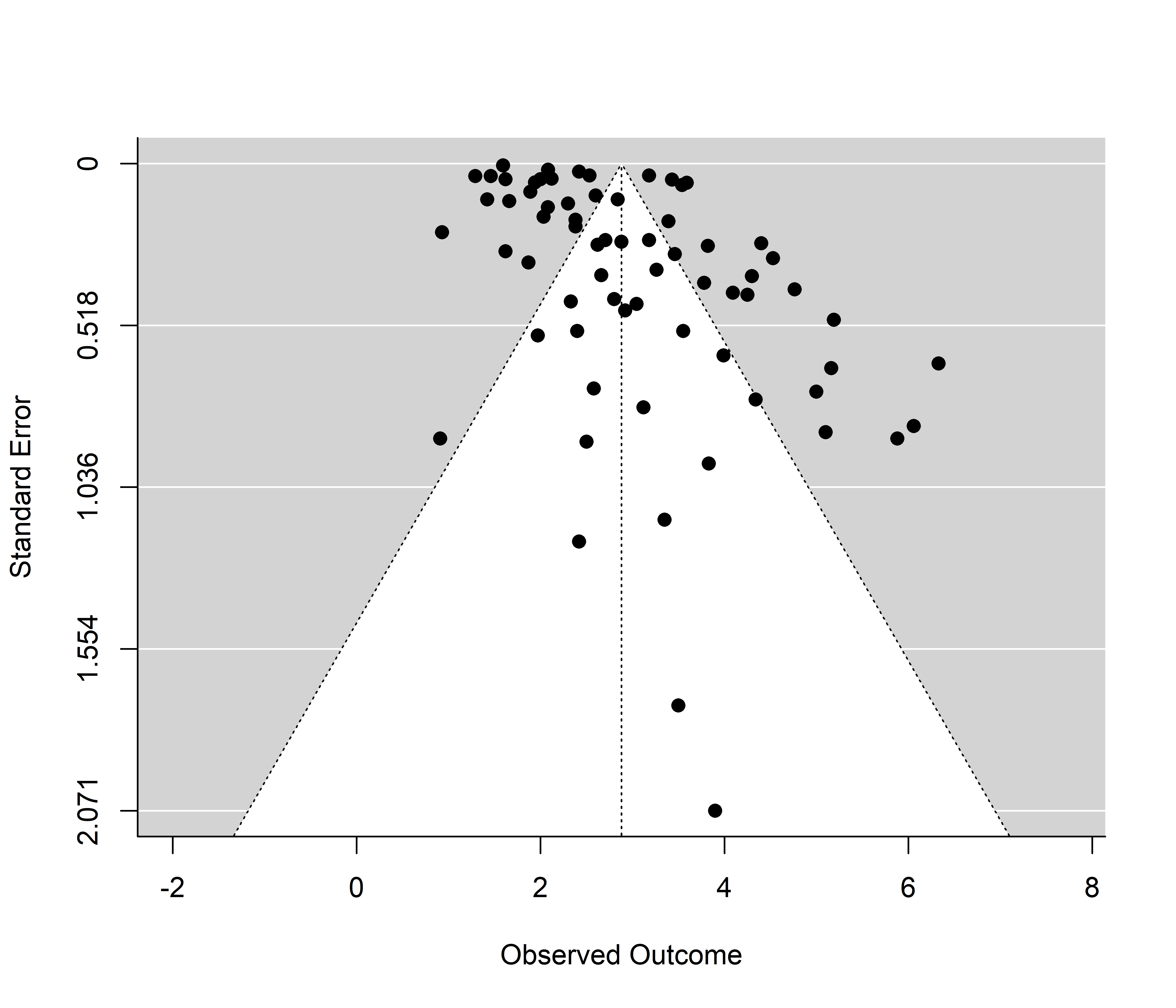


**Table S1 Sensitive analysis for R_0_ using Leave-one-out method**

| Study excluded | R_0_ | se | ci.lb | ci.ub | Q | tau^2^ | I^2^ | H^2^ |
| --- | --- | --- | --- | --- | --- | --- | --- | --- |
| 1 | 2.896638 | 0.096523 | 2.707456 | 3.08582 | 7017.321 | 0.466233 | 99.05947 | 106.323 |
| 2 | 2.867228 | 0.094022 | 2.682948 | 3.051508 | 7686.097 | 0.440215 | 99.14131 | 116.456 |
| 3 | 2.875508 | 0.09415 | 2.690978 | 3.060039 | 7680.429 | 0.440283 | 99.14067 | 116.3701 |
| 4 | 2.909328 | 0.095148 | 2.722842 | 3.095814 | 4053.991 | 0.45034 | 98.37197 | 61.4241 |
| 5 | 2.910398 | 0.094576 | 2.725033 | 3.095764 | 7705.361 | 0.444133 | 99.14345 | 116.7479 |
| 6 | 2.900079 | 0.095639 | 2.712629 | 3.087528 | 7658.099 | 0.456038 | 99.13817 | 116.0318 |
| 7 | 2.921393 | 0.103443 | 2.718649 | 3.124138 | 7432.11 | 0.550327 | 99.11196 | 112.6077 |
| 8 | 2.879559 | 0.093553 | 2.696199 | 3.06292 | 7713.097 | 0.441511 | 99.14431 | 116.8651 |
| 9 | 2.853008 | 0.093759 | 2.669243 | 3.036773 | 7606.896 | 0.435979 | 99.13237 | 115.256 |
| 10 | 2.886992 | 0.094009 | 2.702738 | 3.071245 | 7712.673 | 0.441614 | 99.14427 | 116.8587 |
| 11 | 2.890692 | 0.094361 | 2.705748 | 3.075636 | 7702.198 | 0.442116 | 99.1431 | 116.7 |
| 12 | 2.897486 | 0.094428 | 2.712411 | 3.082561 | 7711.5 | 0.442803 | 99.14414 | 116.8409 |
| 13 | 2.902795 | 0.094277 | 2.718016 | 3.087574 | 7713.858 | 0.442055 | 99.1444 | 116.8766 |
| 14 | 2.889078 | 0.094697 | 2.703475 | 3.074681 | 7291.511 | 0.445234 | 99.09484 | 110.4774 |
| 15 | 2.886665 | 0.09435 | 2.701743 | 3.071587 | 7646.041 | 0.441504 | 99.13681 | 115.8491 |
| 16 | 2.859262 | 0.09159 | 2.679749 | 3.038775 | 7042.197 | 0.410675 | 99.06279 | 106.7 |
| 17 | 2.888837 | 0.094096 | 2.704412 | 3.073262 | 7712.431 | 0.441666 | 99.14424 | 116.855 |
| 18 | 2.879244 | 0.09352 | 2.695948 | 3.06254 | 7713.036 | 0.441503 | 99.14431 | 116.8642 |
| 19 | 2.86829 | 0.093791 | 2.684464 | 3.052117 | 7702.422 | 0.440952 | 99.14313 | 116.7034 |
| 20 | 2.858444 | 0.093933 | 2.674337 | 3.04255 | 7664.28 | 0.438989 | 99.13886 | 116.1255 |
| 21 | 2.861724 | 0.093766 | 2.677946 | 3.045501 | 7694.394 | 0.440493 | 99.14223 | 116.5817 |
| 22 | 2.917569 | 0.095956 | 2.729498 | 3.10564 | 7574.463 | 0.45968 | 99.12865 | 114.7646 |
| 23 | 2.916543 | 0.094284 | 2.731751 | 3.101336 | 7699.894 | 0.44157 | 99.14285 | 116.6651 |
| 24 | 2.878757 | 0.093666 | 2.695174 | 3.06234 | 7712.157 | 0.441474 | 99.14421 | 116.8509 |
| 25 | 2.880577 | 0.094045 | 2.696251 | 3.064903 | 7708.003 | 0.441386 | 99.14375 | 116.7879 |
| 26 | 2.902676 | 0.095712 | 2.715083 | 3.090269 | 7690.672 | 0.456884 | 99.14182 | 116.5253 |
| 27 | 2.879109 | 0.09382 | 2.695226 | 3.062993 | 7711.06 | 0.441446 | 99.14409 | 116.8342 |
| 28 | 2.910481 | 0.095793 | 2.72273 | 3.098233 | 7706.287 | 0.45782 | 99.14356 | 116.7619 |
| 29 | 2.870965 | 0.094091 | 2.68655 | 3.055379 | 7679.695 | 0.440048 | 99.14059 | 116.359 |
| 30 | 2.84472 | 0.093669 | 2.661132 | 3.028308 | 7663.093 | 0.438708 | 99.13873 | 116.1075 |
| 31 | 2.905691 | 0.094592 | 2.720295 | 3.091087 | 7713.421 | 0.444338 | 99.14435 | 116.87 |
| 32 | 2.891992 | 0.094003 | 2.70775 | 3.076234 | 7713.957 | 0.441681 | 99.14441 | 116.8781 |
| 33 | 2.902743 | 0.09533 | 2.715901 | 3.089586 | 7704.956 | 0.45251 | 99.14341 | 116.7418 |
| 34 | 2.861057 | 0.093946 | 2.676927 | 3.045188 | 7678.927 | 0.439753 | 99.1405 | 116.3474 |
| 35 | 2.88386 | 0.093772 | 2.70007 | 3.06765 | 7713.411 | 0.441566 | 99.14435 | 116.8699 |
| 36 | 2.895396 | 0.093776 | 2.711599 | 3.079192 | 7713.173 | 0.441554 | 99.14432 | 116.8663 |
| 37 | 2.883266 | 0.093654 | 2.699707 | 3.066825 | 7713.806 | 0.441566 | 99.14439 | 116.8758 |
| 38 | 2.897058 | 0.094492 | 2.711858 | 3.082258 | 7708.845 | 0.443321 | 99.14384 | 116.8007 |
| 39 | 2.859163 | 0.093668 | 2.675576 | 3.042749 | 7692.175 | 0.440341 | 99.14199 | 116.5481 |
| 40 | 2.885687 | 0.094247 | 2.700967 | 3.070408 | 7703.137 | 0.44152 | 99.14321 | 116.7142 |
| 41 | 2.864418 | 0.093714 | 2.680742 | 3.048093 | 7699.013 | 0.440739 | 99.14275 | 116.6517 |
| 42 | 2.856416 | 0.093667 | 2.672831 | 3.04 | 7687.888 | 0.440099 | 99.14151 | 116.4832 |
| 43 | 2.915386 | 0.096394 | 2.726458 | 3.104315 | 7657.331 | 0.464753 | 99.13808 | 116.0202 |
| 44 | 2.874967 | 0.093719 | 2.69128 | 3.058653 | 7709.451 | 0.441331 | 99.14391 | 116.8099 |
| 45 | 2.850977 | 0.093807 | 2.667119 | 3.034836 | 7667.016 | 0.438998 | 99.13917 | 116.1669 |
| 46 | 2.902024 | 0.094792 | 2.716235 | 3.087813 | 7711.983 | 0.446473 | 99.14419 | 116.8482 |
| 47 | 2.858463 | 0.089165 | 2.683702 | 3.033224 | 6312.373 | 0.384557 | 98.95443 | 95.64202 |
| 48 | 2.88225 | 0.094083 | 2.697852 | 3.066649 | 7708.343 | 0.441438 | 99.14378 | 116.7931 |
| 49 | 2.881395 | 0.094165 | 2.696835 | 3.065955 | 7625.289 | 0.439478 | 99.13446 | 115.5347 |
| 50 | 2.855985 | 0.089943 | 2.679701 | 3.032269 | 6631.637 | 0.392837 | 99.00477 | 100.4794 |
| 51 | 2.872889 | 0.093958 | 2.688734 | 3.057045 | 7702.933 | 0.441051 | 99.14318 | 116.7111 |
| 52 | 2.870699 | 0.094012 | 2.686439 | 3.05496 | 7635.764 | 0.43821 | 99.13565 | 115.6934 |
| 53 | 2.8632 | 0.09397 | 2.679023 | 3.047377 | 7682.222 | 0.43995 | 99.14087 | 116.3973 |
| 54 | 2.852728 | 0.093858 | 2.66877 | 3.036687 | 7658.311 | 0.438582 | 99.13819 | 116.035 |
| 55 | 2.864026 | 0.093985 | 2.679818 | 3.048233 | 7652.884 | 0.438599 | 99.13758 | 115.9528 |
| 56 | 2.852548 | 0.093823 | 2.668658 | 3.036438 | 7630.406 | 0.43716 | 99.13504 | 115.6122 |
| 57 | 2.878821 | 0.094055 | 2.694476 | 3.063165 | 7705.908 | 0.441282 | 99.14351 | 116.7562 |
| 58 | 2.85766 | 0.093781 | 2.673853 | 3.041467 | 7687.078 | 0.440089 | 99.14142 | 116.4709 |
| 59 | 2.869017 | 0.093883 | 2.68501 | 3.053023 | 7700.905 | 0.4409 | 99.14296 | 116.6804 |
| 60 | 2.884215 | 0.094246 | 2.699496 | 3.068934 | 7699.154 | 0.441367 | 99.14276 | 116.6539 |
| 61 | 2.874863 | 0.09411 | 2.69041 | 3.059315 | 7694.536 | 0.440775 | 99.14225 | 116.5839 |
| 62 | 2.890499 | 0.094336 | 2.705605 | 3.075394 | 7704.607 | 0.442003 | 99.14337 | 116.7365 |
| 63 | 2.881024 | 0.094213 | 2.696371 | 3.065678 | 7693.995 | 0.44104 | 99.14219 | 116.5757 |
| 64 | 2.897812 | 0.094236 | 2.713113 | 3.082511 | 7713.981 | 0.441952 | 99.14441 | 116.8785 |
| 65 | 2.892816 | 0.09447 | 2.707657 | 3.077975 | 7696.096 | 0.443007 | 99.14242 | 116.6075 |
| 66 | 2.85478 | 0.090545 | 2.677314 | 3.032245 | 6814.087 | 0.399326 | 99.03142 | 103.2437 |
| 67 | 2.883905 | 0.093867 | 2.69993 | 3.06788 | 7712.804 | 0.441558 | 99.14428 | 116.8607 |
| 68 | 2.884573 | 0.094159 | 2.700024 | 3.069121 | 7707.74 | 0.441505 | 99.14372 | 116.7839 |
